# Cruzipain Sulfotopes-Specific Antibodies Generate Cardiac Tissue Abnormalities and Favor *Trypanosoma cruzi* Infection in the BALB/c Mice Model of Experimental Chagas Disease

**DOI:** 10.3389/fcimb.2021.814276

**Published:** 2022-01-04

**Authors:** Luciana L. Soprano, Maximiliano R. Ferrero, Malena Landoni, Gabriela A. García, Mónica I. Esteva, Alicia S. Couto, Vilma G. Duschak

**Affiliations:** ^1^ Area of Biochemistry of Proteins and Glycobiology of Parasites, Research Department, National Institute of Parasitology “Dr. Mario Fatala Chaben”, ANLIS-Malbrán, Health Department, Ciudad Autónoma de Buenos Aires (CABA, 1063), Buenos Aires, Argentina; ^2^ Organic Chemistry Department, Natural and Exact Sciences Faculty; Research Center in Carbohydrates (CIHIDECAR), University of Buenos Aires, Buenos Aires, Argentina; ^3^ Ministry of Science, Technology and Innovation, National Council of Scientific and Technical Research (CONICET), Buenos Aires, Argentina

**Keywords:** *Trypanosoma cruzi*, cruzipain, C-T domain, sulfotopes, cruzipain sulfotope-specific antibodies, Chagas disease, infection, immunopathogenesis

## Abstract

*Trypanosoma cruzi* cruzipain (Cz) bears a C-terminal domain (C-T) that contains sulfated epitopes “sulfotopes” (GlcNAc6S) on its unique N-glycosylation site. The effects of *in vivo* exposure to GlcNAc6S on heart tissue ultrastructure, immune responses, and along the outcome of infection by *T. cruzi*, were evaluated in a murine experimental model, BALB/c, using three independent strategies. First, mice were pre-exposed to C-T by immunization. C-T-immunized mice (C-T_IM_) showed IgG2a/IgG1 <1, induced the production of cytokines from Th2, Th17, and Th1 profiles with respect to those of dC-T_IM_, which only induced IL-10 respect to the control mice. Surprisingly, after sublethal challenge, both C-T_IM_ and dC-T_IM_ showed significantly higher parasitemia and mortality than the control group. Second, mice exposed to BSA-GlcNAc6S as immunogen (BSA-GlcNAc6S_IM_) showed: severe ultrastructural cardiac alterations while BSA-GlcNAc_IM_ conserved the regular tissue architecture with slight myofibril changes; a strong highly specific humoral-immune-response reproducing the IgG-isotype-profile obtained with C-T_IM_; and a significant memory-T-cell-response demonstrating sulfotope-immunodominance with respect to BSA-GlcNAc_IM_. After sublethal challenge, BSA-GlcNAc6S_IM_ showed exacerbated parasitemias, despite elevated IFN-γ levels were registered. In both cases, the abrogation of ultrastructural alterations when using desulfated immunogens supported the direct involvement of sulfotopes and/or indirect effect through their specific antibodies, in the induction of tissue damage. Finally, a third strategy using a passive transference of sulfotope-specific antibodies (IgG-GlcNAc6S) showed the detrimental activity of IgG-GlcNAc6S on mice cardiac tissue, and mice treated with IgG-GlcNAc6S after a sublethal dose of *T. cruzi*, surprisingly reached higher parasitemias than control groups. These findings confirmed the indirect role of the sulfotopes, *via* their IgG-GlcNAc6S, both in the immunopathogenicity as well as favoring *T. cruzi* infection.

## 1 Introduction

Chagas disease (ChD) is a main health problem caused by the parasitic protozoan *Trypanosoma cruzi*, endemically spread in vast areas of Latin America. There are 6–7 million people infected globally estimated by the World Health Organization (WHO), and further seventy million people are at risk. This illness is endemic in twenty-one countries in the Americas, although the migration of infected people can transport it to endemic and nonendemic countries in the world (Marin Neto et al., 2007; World Health Organization, 2021). Chagas-associated heart disease develops in 10% to 30% of infected individuals and is a common cause of fatal dilated cardiomyopathy (PAHO, Pan American Health Organization, 2021) The complex life cycle of *T. cruzi* contains proliferative stages in the Reduviidae invertebrate vector host (epimastigotes) and in the vertebrate host (intracellular amastigotes), in addition to nonproliferative infectious stages (trypomastigotes) in both hosts (De Souza, 2002). Although the parasite role has been widely accepted during the acute phase of the disease, the participation of *T. cruzi* in the chronic pathogenesis is controversial. There is disagreement regarding the factors that drives the pathogenesis of ChD, but irrespective of the eventual contribution of an autoimmune component (Leon and Engman, 2003; Cunha-Neto et al., 2006), the sustained pathology has been associated with the persistence of *T. cruzi* parasites in the affected organs where they induce chronic inflammation (Tarleton, 2001). Moreover, parasite persistence in conjunction with immune responses against multiple myocardial self-antigens might contribute to chronic heart damage (Girones and Fresno, 2003; Girones et al., 2007). Independently of the proposed causes of the pathogenesis, the severity of chronic ChD has shown to be related with the humoral immune response against epitopes expressed on cruzipain (Cz), the major cysteine protease of this parasite (Duschak et al., 2001a).

In *T. cruzi*, Cz appears as one of the main proteins displaying particularities as antigen, protease, and glycoprotein, which has been extensively studied in the last two decades (Duschak and Couto, 2009). This lysosomal enzyme bears an unusual and highly immunogenic C-T. Although the role of C-T domain is not yet known, it is responsible for most antibodies in natural and experimental infections; thus, it may protect the essential catalytic activity of Cz from antibody opsonization, ensuring parasite survival (Martínez et al., 1993; Del Nery et al., 1997; Cazorla et al., 2010). This domain contains the majority of the posttranslational modifications of Cz. Among them, both the presence of several O-glycosylation sites and the only one N-glycosylation site in the C-T of Cz have been shown. The existence of the O-glycosylation showed O-GlcNAc units (Barboza et al., 2003; Barboza et al., 2005). We have also provided evidence indicating that O-GlcNAc moieties constitute a peculiar common epitope between Cz and either myosin or other cardiac O-GlcNAc-containing proteins, involved in the molecular immunopathogenesis of Chagas heart disease (Acosta et al., 2011). In addition, matrix-assisted ultraviolet laser desorption/ionization time-of-flight mass spectrometry (UV-MALDI-TOF-MS) analysis allowed us to identify the presence of sulfated high-mannose-type oligosaccharides on the unique N-glycosylation site of the C-T (Asn 255) as a new striking feature of this molecule (Barboza et al., 2005). Interestingly, while in virus and mammals sulfated oligosaccharides participate in recognition processes, in *T. cruzi*, we have shown for the first time their involvement as antigenic structures in specific immune responses to Cz (Acosta et al., 2008).

Cz has been related to parasite metabolism (Klemba and Goldberg, 2002) and identified both as an important candidate for vaccine development (Cazorla et al., 2009; Cerny et al., 2016) or forming part of a novel chimerical antigen rationally designed to display B and T cell epitopes among key parasitic protein targets (Bivona et al., 2020) as well as a promising drug target for chemotherapy of ChD (Duschak, 2016; Duschak, 2019).

Data from our laboratory have identified the presence of sulfate-bearing glycosylations in the high immunogenic C-T domain as targets of specific immune responses both in BALB/c model and in chronic ChD patients (Acosta et al., 2008; Couto et al., 2012). We have shown that sulfated moieties are responsible for eliciting IgG2b isotype murine responses to Cz and that subjects chronically infected with *T. cruzi* mount specific humoral immune responses to sulfated glycoproteins. Particularly, to our knowledge, this was the first study demonstrating that C-T, the antigenic domain of Cz, used as immunogen, generates ultrastructural abnormalities in cardiac muscle tissue (Acosta et al., 2008).

We have demonstrated that a glucosamine containing an esterifying sulfate group in position O-6 and an *N*-acetyl group was the preferred epitope for the immunorecognition of sera specific for Cz and its C-T. In addition, in the context of natural infection, immune assays performed with ChD serum confirmed that the structure of synthetic anionic sugar conjugates containing *N*-acetyl d-glucosamine-6-sulfate (GlcNAc-6-SO3) mimics the *N*-glycan-linked sulfated epitope displayed in the C-T of natural Cz (Couto et al., 2012). Interestingly, human IgG2 antibody levels specific for sulfated structures (IgG2-SO_3_) on Cz are inversely correlated with the severity of ChD, indicating that antibodies specific for sulfated moieties might play a relevant biomarker role in the progression of Chagas heart disease (Acosta et al., 2008; Couto et al., 2012).

With the aim to demonstrate the role of the sulfotopes “GlcNAc-6-SO3” (GlcNAc6S) present in the C-T of Cz, in the pathogenesis of ChD, different approaches were conducted on the murine experimental model BALB/c, combining either exposure to sulfotopes by immunization or passive transference of their sulfo-specific IgG (IgG-GlcNAc6S); with or without *T. cruzi* infection. Herein, we have demonstrated for the first time the harmful activity of IgG-GlcNAc6S in cardiac muscle ultrastructure and the input of IgG-GlcNAc6S in the parasite-host interplay favoring *T. cruzi* infection.

## 2 Methods

### 2.1 Parasites and Culture

Epimastigotes of *T. cruzi*, Tulahuen strain, Tul 2 stock (Tul 2), were grown in axenic medium, harvested and washed with 0.25 M sucrose and 5 mM KCl as previously described (Cazzulo et al., 1985). Bloodstream trypomastigotes were *in vivo* maintained by serial passage of blood-form trypomastigotes in BALB/c mice.

### 2.2 Purification of Antigen

Cz purification was carried out from epimastigotes by concanavalin-A (Con-A)-Sepharose affinity column followed by a Mono Q anion exchange column in a fast-performance liquid chromatography (FPLC) system with some modifications (Acosta et al., 2008). The determination of enzymatic activity was performed spectrophotometrically at 410 nm using benzoyl-prolyl-phenyl-alaninyl-*p*-nitro-anilide (Bz-PFA-pNA) as chromogenic peptidyl substrate at pH 8 (Duschak et al., 2003). Inactivation of Cz was performed with 20 µM trans-epoxy-succinyl-L leucyl-amido-4-guanidine butane (E-64) for 1 h on ice. To obtain the purified C-T from Cz, autoproteolysis of the enzyme followed by gel filtration in a BioGel P-30 column was carried out. Elution was monitored by measuring the absorbance at 280/230 nm. To analyze a sample from each fraction, SDS-PAGE followed by electroblotting, using an anti-Cz polyclonal antibody for developing was used (Barboza et al., 2003). C-T protein content was determined in accordance with Lowry’s method (Lowry et al., 1951).

### 2.3 SDS-PAGE With or Without Gelatin as Substrate

Purifications were monitored by 10% SDS-PAGE using the discontinuous buffer system described by Laemmli (1970), and acrylamide mini-gels were stained with silver nitrate (Oakley et al., 1980). Detection of protease activity of purified Cz was performed in 10% resolving SDS-PAGE containing 0.15% co-polymerized gelatin as previously described (Duschak et al., 2001b). Protein content measurement was defined by Bradford´s method (Bradford et al., 1976).

### 2.4 Desulfation Treatments

For chemical desulfation, after samples passage through 0.5 ml of AG50W-X8 resin (H+), the column was washed with water. After the addition of pyridine, the sample was lyophilized, dissolved in dimethylsulfoxide: methanol (9:1 v/v), adjusted to pH 4 with diluted HCl, heated at 100 °C and freeze dried (Freeze et al., 1983).

### 2.5 Coupling of Epitopes to BSA: BSA-GlcNAc6S and BSA-GlcNAc

Immunogens were obtained by coupling commercial GlcNAc6S and GlcNAc to BSA through a glutaraldehyde linkage. GlcNAc6S and GlcNAc were treated with ammonium carbamate in methanol to obtain the 1-deoxy-1-amino sugars, which were used without further purification (Hackenberger et al., 2005). For the coupling reaction, a solution of BSA in phosphate buffer was incubated with the corresponding amino sugar and glutaraldehyde overnight with stirring. The solutions were then dialyzed and desalted for UV-MALDI-TOF MS analysis (Pourcelot et al., 2011).

### 2.6 Immunization of BALB/c Mice and Trypomastigotes Challenge

**Figure 1:**
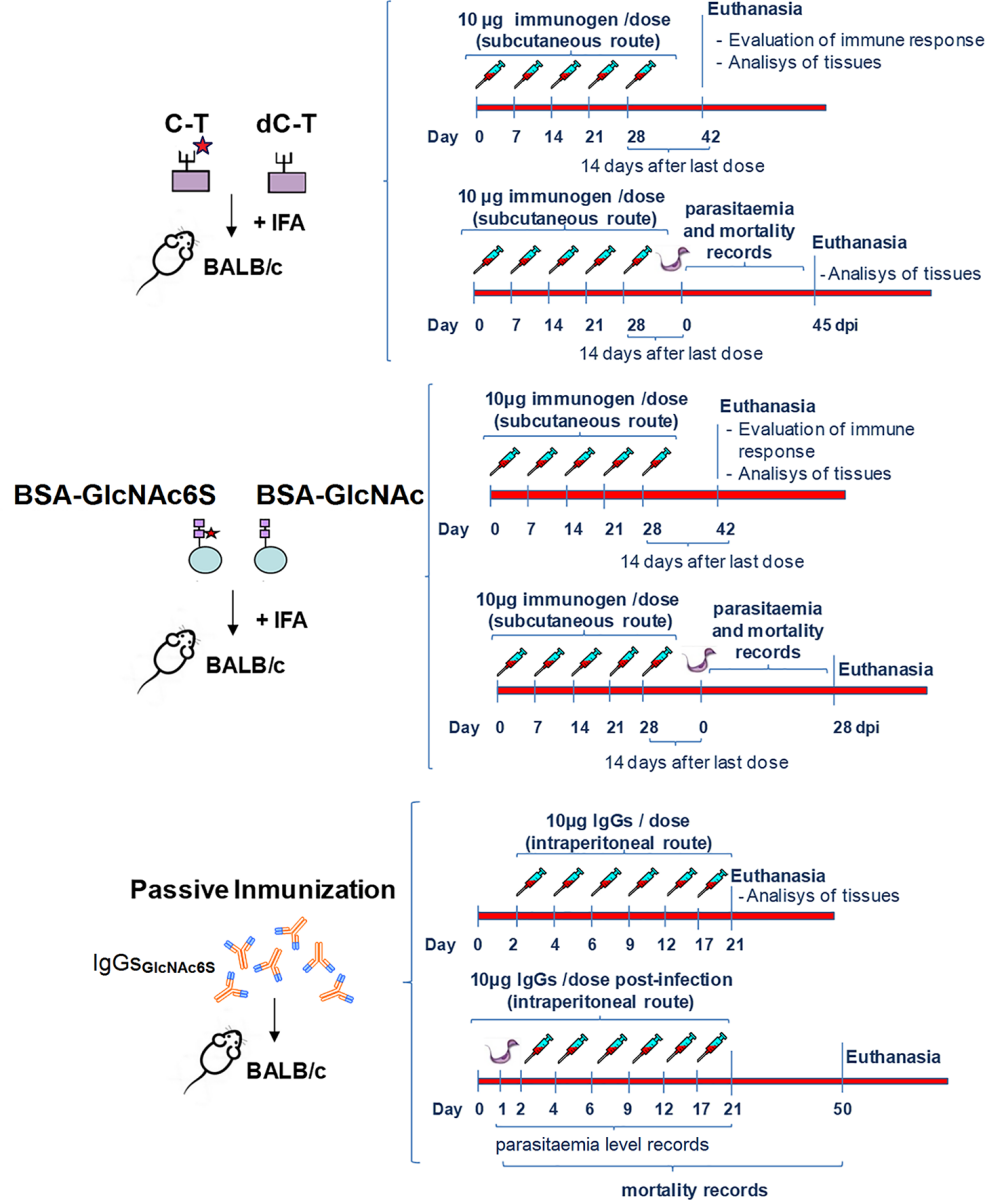
General scheme of immunization from BALB/c mice to study: the input of sulfotopes (GlcNAc6S) in cardiac muscle tissue immunopathology and immune responses, in absence of *T. cruzi* infection, and the participation of the GlcNAc6S during the infection by *T. cruzi.* The scheme details the immunization plans performed with the different immunogens and the passive transference of IgG-GlcNAc6S. The immunization scheme with C-T and dC-T was performed in BALB/c female mice (6–8 weeks old; *n* = 10 animals per group). Five weekly doses of C-T or dC-T (10 μg each), emulsified with IFA (v/v, 1/1) were subcutaneously administered. Control groups received injections with either PBS or with PBS-containing reaction media for solvolysis and were all subjected to the same procedure conditions than the desulfated immunogens plus IFA (Acosta et al., 2008). The immunization scheme with BSA-GlcNAc and BSA-GlcNAc6S was also performed in BALB/c female mice (6–8 weeks old; *n* = 12 animals per group). Five weekly doses of BSA-GlcNAc or BSA-GlcNAc6S (10 μg each), emulsified with IFA (v/v, 1/1) were subcutaneously administered. Control group mice were immunized with BSA+IFA following the same scheme. In both schemes of the immunization, half of the immunized animals with C-T/dC-T; BSA-GlcNAc6S/BSA-GlcNAc and the respective controls were euthanized to evaluate the immune response and perform a tissue analysis, while the other half of immunized mice were submitted to sublethal challenge with 2 × 10^3^ trypomastigotes from Tulahuen 2 (Tul 2) strain, 14 days after the last immunization dose, respectively. Parasitemia was determined each 2–3 days up to negative counting in all groups, and deaths were daily recorded. All the survived mice were subjected to euthanasia in each scheme. For passive transference of antibody assay, BALB/c female mice (6–8 weeks old) were separated in three groups (*n* = 5 mice per group) and received 6 doses of 10 μg from purified IgGs; each were administered by intraperitoneally in days 2, 4, 6, 9, 12, and 17. The groups were treated with IgG-GlcNAc6S, purified from mice sera of BSA-GlcNAc6S_IM_, and purified IgG-pre-imm. Control groups received Phy-Sol administered intraperitoneally. On the other hand, BALB/c female mice (6–8 weeks old) were separated in three groups (*n* = 5 mice per group) and were intraperitoneally infected with 500 *T. cruzi* blood trypomastigotes, Tul 2 strain, the day before to the start of the passive administration for the antibody scheme. A total of 6 doses of 10 μg from purified IgGs each were administered intraperitoneally in days 2, 4, 6, 9, 12, and 17. The mice groups were treated with IgG-GlcNAc6S (IgG-GlcNAc6S-_TM_), IgG-pre-imm (IgG-pre-imm-_TM_), and Phy-Sol. Control groups received Phy-Sol administered intraperitoneally. Parasitemia was monitored each 2–3 days until the fourth day after the last dose of IgGs was administered. Deaths were daily recorded. Euthanasia was performed on days 21 and 50 for tissue analysis and survival recording, respectively. The sublethal infective doses have been selected both by previous experience in the laboratory group and based on bibliography (Lu et al., 2008).

Female BALB/c mice (6–8 weeks old; ten animals per group) were injected subcutaneously with inactive purified C-T, both prior and after chemical desulfation treatment and emulsified with Freund’s incomplete adjuvant (IFA). The first strategy included an immunization protocol that consisted in five weekly doses of 10 µg of each immunogen/mouse/dose. Three groups of controls that received injections with either phosphate buffer saline (PBS) or with PBS containing buffer reaction media for solvolysis (BS), and the infection control group containing physiologic solution (Phy-Sol) were included. All of them were subjected to the same procedure conditions than the desulfated immunogens. PBS and BS were administrated plus IFA (Acosta et al., 2008). In the strategy 2, BALB/c female mice (6–8 weeks old; *n* = 12 animals per group) were exposed to BSA-GlcNAc and BSA-GlcNAc6S, as shown in the immunization scheme. Five weekly doses of BSA-GlcNAc or BSA-GlcNAc6S (10 μg each), emulsified with IFA (v/v, 1/1) were subcutaneously administered. In this strategy, two groups of controls, mice that received BSA in Phy-Sol (BSA + IFA) following the same scheme and the infection control group that only received Phy-Sol during the immunization protocol, were incorporated. In both schemes of the immunizations, half of the immunized animals with C-T/dC-T (C-T_IM_/dC-T_IM_), with BSA-GlcNAc/BSA-GlcNAc6S (BSA-GlcNAc6S_IM_/BSA-GlcNAc_IM_) and the respective controls were euthanized to evaluate the immune response and to perform tissue analysis while the other half of immunized mice were subjected to sublethal challenge with 2 × 10^3^ trypomastigotes from Tul 2 strain 14 days after the last immunization dose (day 42). Parasitemia was determined by direct light microscopy twice a week up to negative counting in all groups. Counts are given as parasite number per milliliter of peripheral blood. Deaths were daily recorded. Survived mice were euthanized 45 and 28 days after the infection, in each scheme, respectively. Also, in strategy 2, serological levels of IFN-γ, expressed as means of each group (pg/ml), were determined weekly up to 28 days postinfection (dpi) by capture ELISA. The third approach consisted in a passive transference of antibody assay with or without *T. cruzi* infection. BALB/c female mice (6–8 weeks old) were separated in six groups (*n* = 5 mice per group). Three of the groups only received purified IgGs, and the other three groups were intraperitoneally infected with 500 *T. cruzi* blood trypomastigotes, Tul 2 strain, the day before the start of the passive administration in accordance with the antibody scheme. Antibody schemes consisting of 6 doses of 10 μg from purified IgGs each were administered intraperitoneally in days 2, 4, 6, 9, 12, and 17. The groups were treated with IgGs specific for GlcNAc6S (IgG-GlcNAc6S), purified from sera of mice immunized with BSA-GlcNAc6S and IgGs purified from sera of preimmune mice (IgG-pre-imm). The control group received Phy-Sol in the times established during the treatment protocol. Parasitemia was monitored as above described, until the fourth day after the last dose of administered IgGs (day 21). Deaths were daily recorded, and euthanasia was performed on day 50. The sublethal infective doses have been selected both by previous experience in the laboratory group and based on bibliography (Lu et al., 2008). The immunization schemes used in each case and the passive administration of specific antibodies are resumed (**Figure 1**). It is worth mentioning that in any case, no significant differences are observed among the control groups’ intra-assay, the graphics show treated vs. controls without discrimination among different controls used. Thus, the graphed control represents all the controls performed. For each strategy, results are representative of three independent experiments.

### 2.7 Anesthesia and Euthanasia

Mice were anesthetized (ketamine 100 mg/kg + xylazine 10 mg/kg, IP), bled by retroorbital sinus, and euthanized by cervical dislocation.

Mice were restrained on a firm, flat surface. The base of the tail was held firmly with one hand, and the back of the neck was fixed at the base of the skull with the thumb and forefinger of the other hand. The dislocation occurred by rapidly pushing forward and down with the hand holding the head while pulling back with the hand holding the base of the tail. The separation of the occipital condyles and the first cervical vertebra was verified by palpation. Confirmation of death was verified by the absence of a heartbeat by palpation and the lack of breathing, corneal reflex, and response to firm toe pinch.

### 2.8 Purification of IgGs

Purified IgGs used in passive transference of antibodies from immunized animals was carried out in accordance with a standard protocol (Ey et al., 1978).

### 2.9 Determination of IgG Isotypes by Enzyme-Linked Immunosorbent Assay

Antibodies specific for purified Cz/dCz or C-T/dC-T on behalf of further analysis of IgG1/IgG2a ratio and isotypes specific for the BSA-GlcNAc6S, and their respective controls, were determined by an indirect ELISA procedure using the corresponding antigens in plate as described in each figure (Duschak et al., 2001a). Briefly, flat bottom (96 wells) plates (Nunc, Thermo Fisher Scientific, Waltham, MA, USA) were coated overnight with 50 µl/well of a solution containing 20 µg/ml of PBS buffer of each antigen. After washing with PBS-Tween 20 (PBS-T), they were blocked using PBS-skimmed milk, washed with PBS-T each, and followed by incubation with a 1:100 dilution of mice sera in PBS-skimmed milk. After repeated washing with PBS-T, specific total IgG was detected by incubation with horseradish-peroxidase-conjugated (HRP) affiniPure goat anti-mouse IgG (H+L) (Jackson ImmunoResearch Laboratories, Inc., West Grove, PA, USA) with a 1:3,000 dilution. After 1 h of incubation, washings were repeated in PBS-T. The reaction was developed with orthophenylenediamine (OPD) as substrate in darkness, using citrate buffer pH 5 in the presence of hydrogen peroxide at 30%. Samples were assessed in triplicates and were stopped with 1 M sulfuric acid. Color was measured at 490 nm with an automatic ELISA reader. Specific IgG isotypes were detected by the same ELISA indirect procedure as that of total IgG, but a 1:200 dilution of biotinylated murine anti-mouse IgG1, IgG2a, and IgG2b was used (PharMingen, BD Biosciences, Franklin Lakes, NJ, USA), followed by incubation with streptavidin-HRP. IgG2a/IgG1 ratios were calculated to be used as indicator of Th1 or Th2 in the immune responses induced by Cz and C-T with and without desulfation treatment (Acosta et al., 2008).

### 2.10 Cellular Immune Response in BSA-GlcNAc6S-Immunized BALB/c Mice Strain

#### 2.10.1 Delayed-Type Hypersensitivity Measurement

Fifteen days after the last dose, cell mediated immune response induced by immunization with BSA-GlcNAc6S, and BSA-GlcNAc was evaluated by delayed-type hypersensitivity (DTH) measurement. BSA-GlcNAc6S, BSA-GlcNAc, and BSA were intradermally inoculated in dorsal metatarsal hind-limb. DTH assays were quantified as a standard method, considering the indurations produced in the dorsal metatarsus of mice 24 and 48 h after intradermal inoculations. The bump thickness and induration diameter were measured with a micrometric caliber (Mitutoyo, Kawasaki, Japan). Control mice were inoculated with all these antigens to take account of nonspecific reactions.

### 2.11 *In Vitro* Culture of Splenocytes and Determination of Cytokines by Flow Cytometry

Handling of spleens obtained by necropsy was performed in sterile conditions. After homogenization, red blood cell lyses were performed by addition of lyses buffer (Sigma Aldrich, St. Louis, MO, USA) and after spleen cells were suspended in Roswell Park Memorial Institute 1640 Medium (RPMI) (Thermo Fisher Scientific, USA) supplemented with 10% bovine fetal serum (BFS), 2% penicillin (100 μg/ml), streptomycin (100 U/ml), and 0.4 mM β-mercaptoethanol. Viable spleen cells were counted by Trypan Blue method in Neubauer chamber and 1 × 10^6^ spleen cells were cultured in 96-well plates (Nunc, Thermo Fisher Scientific, USA), in RPMI alone, or with the stimuli. The stimulation was performed with 10 μg/ml of Cz, dCz, C-T, and dC-T and with RPMI (Thermo Fisher Scientific, USA). A Con-A solution (2 μg/ml) was used as unspecific stimulus to verify the viability of the spleen cells. They were cultured at 37°C and 5% CO_2_ for 72 h. Cytokines were measured by flow cytometry, using the Cytometric Bead Array (CBA) Mouse Th1/Th2/Th17 Cytokine Kit (BD Biosciences, USA). Flow cytometry determinations were performed in accordance with the manufacturer instructions. In addition, it is worth mentioning that the values obtained, using RPMI as stimulus, were discounted in all cases.

### 2.12 Histopathological Analysis Was in Accordance With Traditional Techniques

C-T_IM_ with or without chemical desulfation treatment and control groups were sacrificed after the last immunization dose or after the acute phase of the infection, in accordance with the day described in **Figure 1**. Mice were anesthetized, bled by retroorbital sinus, and euthanized by cervical dislocation. We performed complete necropsies and histopathological analysis on immunized and control mice. After removal of heart and a piece of skeletal muscle from hind-limbs, they were rinsed with PBS and fixed in 10% buffered formalin. Fixed tissues were dehydrated in absolute ethanol, cleared in xylene, and embedded in paraffin. Sections (5 μm) were stained with hematoxylin-eosin and examined by light microscopy. For skeletal and heart muscle tissues, the evaluation was qualitative/descriptive and semiquantitative of the inflammatory infiltrate. For skeletal muscle tissue, the myositis “score” was determined in accordance with the distribution and extension of the inflammatory infiltrate, being 1 = simple focus of inflammatory infiltrate; 2 = multiple nonconfluent foci of inflammatory infiltrate; 3 = confluent inflammation; and 4 = diffusely extended inflammation (Martin et al., 2007). For heart tissue, the following classifications of myocarditis were used: normal tissue, score 0; mild focus, light infiltration with damage of one or two myocardial fibers, score 1; focus of moderate size and inflammatory aggregates comprising 3 to 5 fibers, score 2; and intense foci with thick accumulation of mononuclear cells with destruction of more than 5 muscle fibers, score 3 (Bontempi et al., 2015). Photographs, representative of five sections out of four mice, from each immunization group, were randomly taken from three independent experiments and further analyzed.

### 2.13 Ultrastructural Electron Microscope Analysis

When the necropsy was performed, cardiac muscle samples were extracted from different immunized and control mice groups and fixed by immersion in 3% glutaraldehyde in 0.2 M phosphate buffer, pH 7.4, for 24 h at low temperature. After washing and slicing into small blocks, they were postfixed in OsO_4_, dehydrated in graded alcohol solutions, and embedded in epoxy resins as described previously (Acosta et al., 2008). Thin sections were sliced in a Porter-Blum MT2 Sorvall ultra-microtome and the sections picked up in 300-mesh single-hole grids. Staining was done first with uranyl acetate and then with Reynolds lead citrate stain. The specimens were examined under a Siemens Zeiss C10 (Siemens, Munich, Germany) electron microscope. Photographs were taken with a Kodak electron imaging film (Eastman Kodak, Rochester, NY, USA). This equipment was used in the facilities of Lanais-MIE, Faculty of Medicine, UBA.

### 2.14 Statistical Analysis

Unpaired *t*-test was used in IL-17/IL-10 ratio determinations. The Mann-Whitney nonparametric test was selected for comparisons of one factor between two groups in the analysis of the isotype profile and DTH in BSA-GlcNAc6S_IM_ and BSA-GlcNAc_IM_. Nonparametric Kruskal-Wallis test followed by multiple comparisons of Dunn’s test was selected for both the determination of isotype levels and their IgG2a/IgG1 ratio as well as for cytokine analysis. Statistics was carried out with Prism software version 5.0 (GraphPad, San Diego, CA, USA).

## 3 Results

With the aim to elucidate whether the sulfotopes located in the C-T of Cz play a role in the experimental ChD, the *in vivo* effects of the exposure to GlcNAc6S on ultrastructure of cardiac muscle tissue, immune responses, and the outcome of *T. cruzi* infection were studied in a murine experimental model using three strategies: (1) an immunization with C-T; (2) an immunization to expose the synthetic GlcNAc6S coupled to BSA; and (3) a passive transference treatment of purified IgG-GlcNAc6S prior and/or after sublethal challenge with *T. cruzi*, Tul 2, depending on the approach used (**Figure 1**).

### 3.1 Effects of Immunization With C-T and dC-T on BALB/c Mice: Evaluation of Cardiac Muscle Tissue Ultrastructure, Immune Responses, and Course of *T. cruzi* Infection

No cardiac muscle abnormalities have been observed by optical microscopy using hematoxylin and eosin staining, in C-T_IM_. However, the ultrastructural study of cardiac muscle tissue in C-T_IM_ by electron microscopy revealed anomalies both in myofibrils and mitochondria as we have previously described (Acosta et al., 2008), in absence of infection.

#### 3.1.1 Immune Responses Triggered by Exposition With C-T and dC-T to BALB/c Mice

##### 3.1.1.1 (a) Isotype Sera Levels and IgG2a/IgG1 Bias

**Figure 2:**
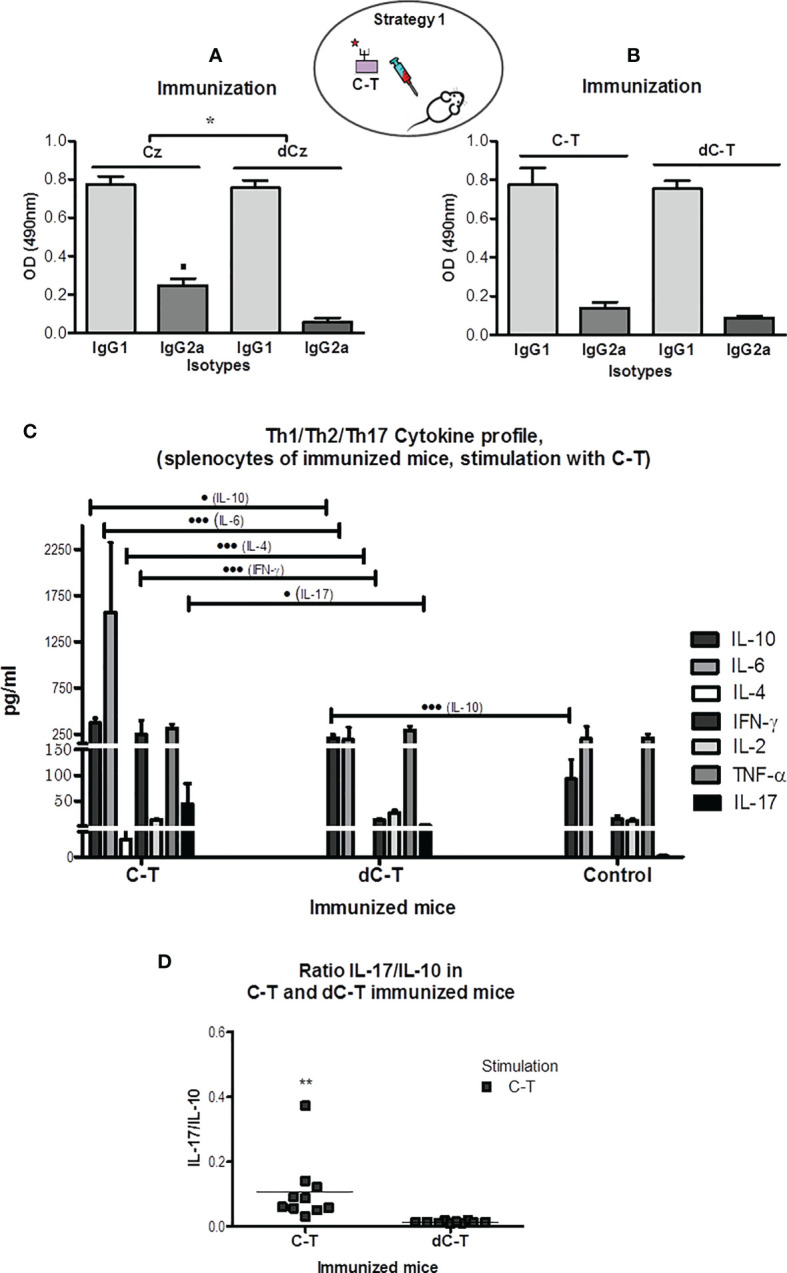
IgG1 and IgG2a humoral immune responses in sera from BALB/c mice exposure of GlcNAc6S by immunization with Cz, C-T, and its desulfated counterparts. Determination of IgG2a/IgG1 ratios. IgG1 and IgG2a isotype levels were measured in sera from Cz_IM_ and dCz_IM_ **(A)** and C-T_IM_ and dC-T_IM_ **(B)** by ELISA. In each case, the antigen adsorbed in the plate is Cz. The bars represent the averages of duplicate determinations, and the error bars indicate the SD. **(A)** ▪ *p* < 0.05 vs. IgG2a dCz. IgG2a/IgG1 ratios ^*^ *p* < 0.001 Cz_IM_ vs. dCz_IM_. **(C)** Flow cytometry measurement of the cytokine levels of IL-10, IL-6, IL-4, IFN-γ, IL-2, TNF-α, and IL-17 in supernatants of spleen cell cultures from C-T_IM_, dC-T_IM_, and control mice, stimulated with C-T. The supernatants were obtained after 48 h of stimulation with C-T (10 μg/ml). The concentration of each cytokine (pg/ml) was obtained with the software BD FCAP Array v3.0. The bars represent the average of six determinations by duplicate with their SD. Production of IL-10: • vs. dC-T_IM_; ••• vs. control mice. Production of IL-6: ••• vs. dC-T_IM_; production of IL-4: ••• vs. dC-T_IM_. Production of IFN-γ: ••• vs. dC-T_IM_. Production of IL-17: • vs. dC-T_IM_. (• *p* < 0.05; •••*P* < 0.001). **(D)** Determination of IL-17/IL-10 ratio in C-T_IM_ and dC-T_IM_, calculated from levels obtained by flow cytometry. The values of the IL-17 and IL-10 concentrations used were those obtained with C-T as stimulant. (^**^ *p* = 0.0083; C-T_IM_ vs. dC-T_IM_). The oval scheme in the upper part of the figure represents strategy 1. IgG2a/IgG1 ratios *p < 0.001 CzIM vs. dCzIM. The symbol means p minor to 0.001.

To evaluate the effect of sulfated moieties on the humoral responses against Cz/C-T, we immunized BALB/c mice with either purified Cz (devoid of enzymatic activity) or purified inactive C-T, in both cases prior (Cz, C-T) (**Figures 2A, B**, left half) and after (dCz, dC-T) (**Figures 2A, B**, right-half) desulfation treatment. The levels of the IgG2a and IgG1 isotypes were determined (**Figures 2, B** **)**, and the ratio IgG2a/IgG1 was used as an indicator of the Th1 or Th2 trend.

All immunizations with Cz, dCz, C-T, and dC-T showed similar and higher levels of IgG1 than IgG2a against Cz in plate. The highest Cz recognition by IgG2a was observed in Cz-immunized mice (**Figure 2A**, left half). The low values obtained for the ratio IgG2a/IgG1 in all immunization groups suggested a Th2-type response against the natural molecule present in the parasite. Mice that were immunized with desulfated molecules presented the lowest ratios. However, significant differences were only observed in Cz-immunized mice (IgG2a/IgG1 = 0.318 ± 0.035) compared with those immunized with dCz (IgG2a/IgG1 = 0.074 ± 0.021).

Regardless of the immunogen used, an unfavorable Th2 polarization was observed for facing up the parasite challenge. However, a sulfotope-specific IgG2a isotype response generated by immunization with Cz suggests a trend towards a more favorable reaction capable of resisting a parasitic infection (Bontempi et al., 2015).

##### 3.1.1.2 (b) Spleen Cell Supernatant Cytokine Levels and Th Profiles

The specific cytokine profile triggered in C-T_IM_ and dC-T_IM_ was quantified through flow cytometry. Cytokines belonging to the Th1 (INF-γ, IL-2, TNF-α), Th2 (IL-6, IL-4, IL-10), and Th17 (IL-17) profiles were determined in supernatants of spleen cells.

To emulate the hypothetical cytokine profile that mice pre-exposed to immunizations with C-T, dC-T, and controls would display to the parasite GlcNAc6S, in absence of infection, purified *T. cruzi* C-T was selected as stimulus.

C-T_IM_ showed significantly higher levels of the three cytokines representing the Th2 profile: IL-10, IL-6, and IL-4 and significant differences in the levels of IFN-γ and IL-17 than those of the dC-T_IM_. By contrast, dC-T_IM_ induced a very similar cytokine profile to that from the control group, except for the significant elevated levels of IL-10, an anti-inflammatory cytokine belonging to the Th2 profile (**Figure 2C**).

After stimulation with C-T, the *in vitro* production of cytokines by splenocytes of C-T_IM_ showed the following: IL-10 levels significantly high in comparison with those from dC-T_IM_ (*p* < 0.05), while the latter were significantly higher than nonimmunized controls (*p* < 0.001); remarkable levels of IL-6, significantly higher than those observed in the dC-T_IM_ (*p* < 0.001); IL-4 production showed significant differences compared with those from dC-T_IM_ (*p* < 0.001); INF-γ presented higher and significant levels than those obtained with either dC-T_IM_ or control group (*p* < 0.001); and levels of IL-17, proinflammatory cytokine, representative from Th17 profile (Gutierrez et al., 2009; Miyazaki et al., 2010; Beringer et al., 2016), showed significant differences in comparison with those of the dC-T_IM_ (*p* < 0.05) (**Figure 2C**). The determination of cytokines from splenocytes of BALB/c mice stimulated with Con-A, as unspecific stimulation control, clearly showed that the capacity of response in the dC-T_IM_ is lower than that observed in the control group, and the C-T_IM_ profile showed to be comparable with that of controls, however, with higher values than the controls. This is in line with our observations when stimulations are performed with sulfated parasitic antigens (**Supplementary Figure S1**). Two-dimensional dot plots corresponding to the cytokines profile of a representative mouse from each immunization group are shown in **Supplementary Figure S2**.

Additionally, the IL-17/IL-10 ratio was calculated. A significant increased value was shown in C-T_IM_ (**Figure 2D**), suggesting that IL-17 exerts a favorable modulator role on cytokines responsible for the resistance to infection, in contrast to the anti-inflammatory prevalence due to IL-10 values recorded in the dC-T_IM_ group. Therefore, IL-17 increase could contribute as a regulatory component of INF-γ levels, in association with IL-10, as cytokine is capable of counteracting the deleterious effects of proinflammatory cytokines in C-T_IM_ (Guedes et al., 2010; Miyazaki et al., 2010). It was observed that the use of Cz as stimulus induced an increased production of IL-17 in C-T_IM_ (**Supplementary Figure S3**). For all other cytokines studied, similar results were obtained using both C-T and Cz as stimuli.

The stimulation with a sulfated parasitic antigen to spleen cells from mice pre-exposed to the GlcNAc6S, by C-T immunization has induced the production of relevant cytokines to face up the challenge with *T. cruzi*, suggesting a proinflammatory/anti-inflammatory balance, modulated by IL-17, potentially favorable to host resistance (Guedes et al., 2010), compared with the group that was not previously exposed to the GlcNAc6S from dC-T_IM_ (Guo, 2016). In summary, C-T_IM_ induces the production of cytokines from Th2, Th17, and Th1 profiles, while dC-T_IM_ only induces IL-10 (Th2) with respect to the control mice.

#### 3.1.2 Effects of C-T Sulfotopes on Parasitemia, Survival, and Tissue Pathogenicity of BALB/c Mice Sublethally Challenged With *T. cruzi*

**Figure 3:**
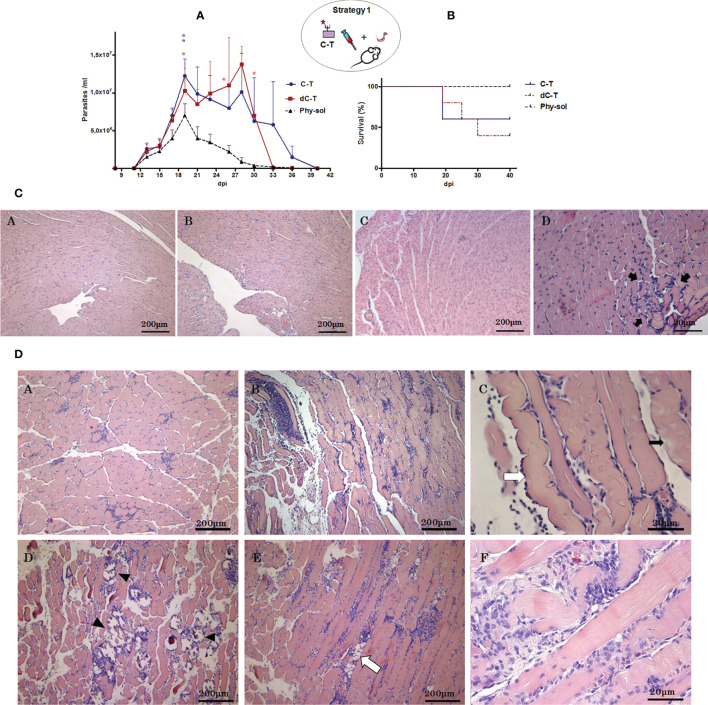
Effects of C-T sulfotopes on parasitemia, survival, and tissue pathogenicity of BALB/c mice sublethally challenged with *T. cruzi*. **(A)** Parasitemia (parasite number/ml of peripheral blood). **(B)** Survival percentage (%) from control, C-T_IM_, and dC-T_IM_ (*n* = 5 mice per group) was recorded after the sublethal challenge with a dose of 2 x 10^3^ blood trypomastigotes of *T. cruzi*, Tul 2 strain. Error bars indicate SD of the means. The days in which deaths were registered were marked in the curve with asterisks (^*^), in red and blue color. Blue lines represent C-T_IM_; red lines, dC-T_IM_; and grey dotted lines, Phy-Sol control group. Histopathologic alterations of cardiac **(C)** and skeletal muscle **(D)** tissues from nonimmunized control, C-T_IM_, and dC-T_IM_, postsublethal challenge by *T. cruzi*. **(C)** Optical microphotographs of cardiac muscle tissue transversal sections stained with hematoxylin/eosin obtained from nonimmunized control (A), dC-T_IM_ (B), and C-T_IM_ (C, D); 45 dpi. In (D), a lymphoplasmacytic inflammatory infiltrate focus is shown with greatest detail between arrows. **(D)** Optical microphotographs of sections from skeletal muscle tissue stained with hematoxylin/eosin obtained from nonimmunized control (A), C-T_IM_ (B), and dC-T_IM_ (D). [(A, B, D): ×10]. Longitudinal section of skeletal muscle fibers of C-T_IM_ (×40) (C), indicated with a white arrow is an edematous muscle fiber and with black arrow lyses foci of the muscle fiber. In the skeletal muscle tissue from dC-T_IM_, dystrophic calcifications (arrow tip) (D) and lipid degeneration (E) (white arrow) can be observed; (D) and (E) at ×10. At 40 x magnification, a diffused severe lymphoplasmacytic inflammatory infiltrate and lyses foci (F) in dC-T_IM_ can be observed. The oval scheme in the upper part of the figure represents strategy 1.

To address the participation of C-T sulfotopes along the course of experimental ChD, 14 days after the last immunization dose with C-T or dC-T (**Figure 1**), BALB/c mice were challenged with a sublethal dose of *T. cruzi* Tul 2 blood trypomastigotes. Parasitemia and mice survival were recorded after the third day postsublethal infection (**Figures 3A, B** **)**. The nonimmunized control group presented a parasitemia peak 19 dpi +1, without mortality registered (100% survival) during the acute phase. By contrast, C-T_IM_ and dC-T_IM_ showed a double parasitemia peak: the first one centered around 19 dpi and the second one is close to 28 dpi. In turn, parasitemia showed a different behavior in both groups. C-T_IM_ presented higher parasite number values during the first peak of parasitemia than during the second one. On the other hand, dC-T_IM_ presented higher parasitemia counting during the second peak than those from the first peak. Area under the curve (AUC) was used to evaluate and compare the parasitemia along the period under study among groups. C-T_IM_ and dC-T_IM_ recorded similar AUC values (3.08 and 2.86 times higher than control, respectively). Both immunized mice groups presented lengthier and higher parasitemia than the control group (AUC: 5.52 × 10^6^/ml). C-T_IM_ was characterized by detectable parasitemias for longer periods of time than that from dC-T_IM_, while this group stood out for the highest parasite/ml counting recorded.

The mortality showed no significant differences among immunized groups; however, C-T_IM_ revealed higher survival than dC-T_IM_, indicating a mortality lower than 50%, which was associated to the highest parasitemia values during the first peak (**Figure 3A, B** **)**. By contrast, deaths in dC-T_IM_ occurred during a more extended period and in association to both parasitemia peaks, resulting in a mortality higher than 50% (**Figures 3A, B** **)**. At difference with the control group, C-T_IM_ and dC-T_IM_ reached values of lethal parasitemia, although they had been challenged with sublethal doses (**Figure 3A**). In addition, knowing that the presence of ultrastructural damage generated in cardiac muscle tissue by exposition to C-T sulfated molecules before challenge with *T. cruzi*, the fact that the C-T_IM_/dC-T_IM_ mortality were not significantly different was also outstanding.

The histopathologic analysis of skeletal and cardiac muscle tissue from mice that surpassed the acute infection was performed. Tissues were classified in accordance with their inflammatory infiltrate. In this way, mild myocarditis was detected in infected controls and dC-T_IM_ while in C-T_IM_ mild to moderate myocarditis was observed (Bontempi et al., 2015) (**Figure 3C**). Severe myositis was noticed in skeletal muscle from both C-T_IM_ and dC-T_IM_, and nonconfluent focal myositis was detected in muscle from control of infection mice (Martin et al., 2007) (**Figure 3D**). The skeletal muscle tissue from both C-T_IM_ and dC-T_IM_ presented a similar inflammatory “score.” However, the histopathological analysis of skeletal muscle of dC-T_IM_ confirmed that this group coursed the acute phase infection with the greatest severity. The skeletal muscle tissue from survival dC-T_IM_ revealed the following in the qualitative histopathological analysis: (i) presence of edematized fibers with loss of the typical transverse striation, (ii) hydropic degeneration or muscular fiber lysis, and (iii) diffuse lymphoplasmocitary inflammatory infiltrate distributed in the interstitial space from the field and surrounding the dystrophic calcification focus. Likewise, the presence of interfibrillar steatosis by degeneration of interstitial connective cells was observed. Many of the mentioned injuries were also observed in C-T_IM_, once the acute phase of the infection was surpassed. However, muscle tissue areas with multiple dystrophic calcification foci, which denote infected muscle fibers followed by necrosis, were notably extended in dC-T_IM_ compared with C-T_IM_. The results observed in skeletal muscle from dC-T_IM_ showed that the immunological response was unable to limit the pathogen virulence. This point is compatible with the parasitemia curve observed. In conclusion, after a *T. cruzi* sublethal dose, survival of mice pre-exposed to GlcNAc6S from C-T_IM_ surpassed an exacerbated parasitemia and presented severe circumscribed skeletal muscle injuries; on the other hand, in absence of pre-exposition to the C-T sulfotopes, dC-T_IM_ showed parasitemia exacerbation, slightly high lethality, and severe extended lesions in the skeletal muscle tissue denoting an immunologic response unable to restrict the pathogen virulence.

### 3.2 Effects Induced by *In Vivo* Pre-Exposition of Synthetic GlcNAc6S Coupled to BSA (BSA-GlcNAc6S) to BALB/c mice, Both on Heart Muscle Tissue and Immune Responses and on the Course of *T. cruzi* Infection

#### 3.2.1 Ultrastructural Effects on Cardiac Muscle Tissue in BALB/c Mice Exposed to BSA-GlcNAc6S

**Figure 4:**
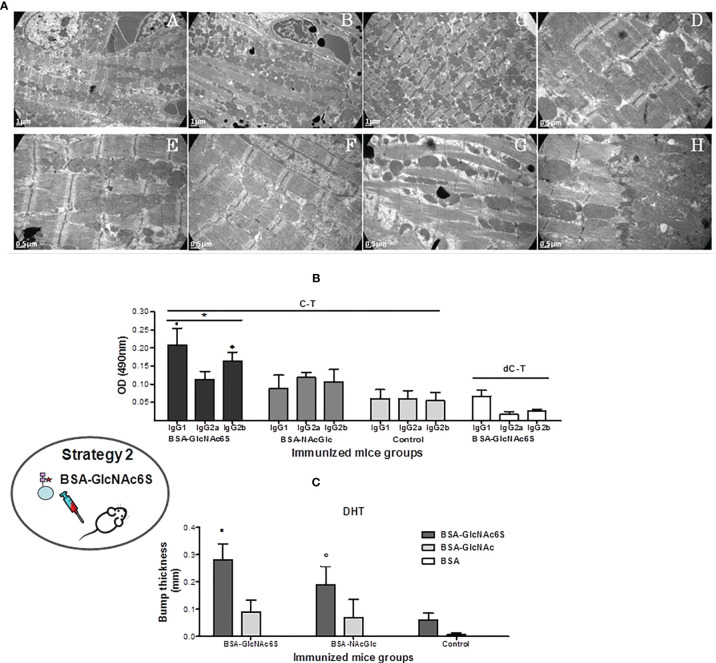
Immunization of BALB/c mice with GlcNAc6S-BSA in the absence of infection. **(A)** Ultrastructural analysis of cardiac tissue of BSA-GlcNAc6S_IM_, BSA-GlcNAc_IM_, and BSA-Phy-Sol_IM_. Cardiac muscle BSA-GlcNAc6S_IM_ showing severe abnormalities (C) in comparison with normal controls BSA-PhySol (A) and BSA-GlcNAc (B) by transmission electron-microscopy. Magnification of muscle tissue longitudinal sections (7000 ×). At a major magnification, ultrastructural alterations were evidenced in a section of BSA-GlcNAc6S_IM_ [20000 × (D, H)12,000 × (G)], in comparison with the micrograph corresponding to BSA-GlcNAc_IM_ (F) with slight changes and to the normal structure in the BSA-PhySol control mice (E). Determination of immune response to synthetic GlcNAc6S. **(B)** Humoral immune response in BSA-GlcNAc6S_IM_. Isotype profile levels in BSA-GlcNAc6S_IM_ and BSA-GlcNAc_IM_ by indirect ELISA, using C-T and dC-T as plate antigens. Control mice were immunized with BSA-IFA. The bars represent the average of the determinations by duplicates and their SD. Black bars, dark grey bars, and light grey bars represent BSA-GlcNAc6S_IM_, BSA-GlcNAc_IM_, and BSA, respectively, on C-T antigen. White bars represent BSA-GlcNAc6S on dC-T antigen. • *p* = 0.0079 vs. IgG2a in BSA-NAcGlc6S_IM_; *p* = 0.0079 vs. IgG1 in BSA-GlcNAc_IM_; ♦ *p* = 0.0159 vs. IgG2b from BSA-GlcNAc_IM_. ^*^C-T vs. dC-T in plate: *p* = 0.0079 IgG1 (C-T) vs. IgG1 (dC-T); *p* = 0.0119 IgG2a (C-T) vs. IgG2a (dC-T); *p* = 0.0079 IgG2b (C-T) vs. IgG2b (dC-T). **(C)** T-dependent cellular response obtained with GlcNAc6S. Delayed type hypersensitivity (DTH) was measured in dorsal metatarsal hind-limb of control (BSA_IM_) (*n* = 5), BSA-GlcNAc6S_IM_ (*n* = 5), and BSA-GlcNAc_IM_ (*n* = 5), 24 and 48 h after intradermal inoculation of BSA-GlcNAc6S(_ID_), BSA-GlcNAc (_ID_), and BSA (_ID_). The bars represent the average of the dorsal metatarsal hind-limb indurations thick with their SD. Dark grey bars, light grey bars, and white bars represent BSA-GlcNAc6S_(ID)_, BSA-GlcNAc_(ID)_, and BSA_(ID)_ intradermic antigen, respectively. ▪ *p* = 0.0079 vs. BSA-GlcNAc (_ID_) in BSA-GlcNAc6S_IM_; ^○^ *p* = 0.0357 vs. BSA-GlcNAc6S (_ID_) in control mice. The oval scheme in the left part of the figure represents strategy 2.

The ultrastructural cardiac tissue analysis of BSA-GlcNAc6S_IM_ showed a severe alteration in the tissue architecture including sections of complete structural myofibrils and mitochondria disorganization without the grouping characteristic pattern in **Figure 4A**.C (at 7,000 ×); increase of the sarcoplasmic space (**Figure 4A**.G); evidence of a remarkable slenderness of cardiac myofibrils and enlargement of sarcomeres (**Figures 4A.**D**, A**.H), and also mitochondria disruption and cristae free in the sarcolemma (**Figure 4A**.H). Despite these severe alterations, intercalary disc integrity was conserved (**Figure 4A**.H). It is worth noting that the absence of band H, which is not observed in **Figures 4A.**C**, A**.G, is due to the contraction state of muscle fibers. In contrast, cardiac tissue from BSA_IM_ control retained a regular pattern of normal cardiac tissue (**Figures 4A.**A**, A**.E) showing regular fibril distribution and grouped mitochondria in parallel to the longitudinal axis of myofibrils, while sarcomeres showed a typical regular pattern of transversal striation. It is worth mentioning that although BSA-GlcNAc_IM_ conserved the regular tissue architecture, it showed a slight myofibril slenderness and enlargement of sarcomeres (**Figures 4A.**B**, A**.F).

#### 3.2.2 Humoral Immune Response in BALB/c Mice Exposed to BSA-GlcNAc6S

To focus into the involvement of the GlcNAc6S located in the C-T from Cz in the humoral immune response, BALB/c mice were immunized with BSA-GlcNAc6S, using BSA as protein carrier, in combination with IFA (**Figure 1**). By immunization with BSA-GlcNAc6S, an IgG isotype profile comparable with that obtained with either Cz or C-T immunizations was determined (Acosta et al., 2008). It means a prevalence of IgG1 isotype with respect to IgG2a and high levels of IgG2b (**Figure 4B**). The analysis of IgG subclasses profile from sera specific for GlcNAc showed low levels in all IgG isotypes, with prevalence of IgG2a, IgG2b, followed by IgG1 when confronted with C-T, as antigen in the plate, in comparison with GlcNAc6S. However, IgG2a sera specific for GlcNAc (IgG2a-GlcNAc) showed similar C-T recognition levels than anti-GlcNAc6S, demonstrating the high participation of the IgG2a-GlcNAc in the unspecific recognition. In mice BSA-GlcNAc6S serum, the isotype profile of dC-T recognition was evaluated showing a significant decrease of IgG1, IgG2a, and IgG2b isotypes (**Figure 4B**), confirming their involvement in the recognition of the GlcNAc6S and demonstrating the specificity of the humoral response obtained for sulfotopes. BSA-GlcNAc6S can trigger qualitatively different humoral response; additionally, the GlcNAc6S has demonstrated to be more immunogenic than BSA-GlcNAc. The tittering of both sera with C-T as antigen showed that the polyclonal anti-BSA-GlcNAc6S serum presented a title (1:102.400) significantly higher than that from anti-BSA-GlcNAc serum (1:25.100). Once the humoral immune responses are known, the cross-reactivity between the immunogens (BSA-GlcNAc6S; BSA-GlcNAc) was evaluated, with either serum specific for BSA-GlcNAc6S or BSA-GlcNAc. No significant differences were found in the recognition of the GlcNAc6S by any of the two specific sera (**Supplementary Figure S4**), indicating a low specificity for IgG-GlcNAc. In conclusion, humoral immune response to sulfotopes (IgG-GlcNAc6S) is shown to be strong, highly specific, and is able to reproduce the same specific IgG isotype profile to that obtained with the C-T exposure.

#### 3.2.3 T-Dependant Response in BALB/c Mice Exposed to BSA-GlcNAc6S

With the aim of evaluating the cellular response generated by the GlcNAc6S, BSA-GlcNAc6S was inoculated in dorsal metatarsal hind-limb of BSA-GlcNAc6S_IM_, BSA-GlcNAc_IM_, and nonimmunized control mice. Significant differences were detected in the induration thickness of mice from the BSA-GlcNAc6S_IM_ compared with those from BSA-GlcNAc_IM_ and from the latter in comparison with the control ones (**Figure 4C**). Significant differences were also observed in the BSA-GlcNAc6S_IM_ when the intradermal (ID) antigen used was BSA-GlcNAc6S instead of BSA-GlcNAc. Even though the GlcNAc6S-BSA has demonstrated a nonspecific cell recruitment capacity in the mice control group, the ID inoculation with the GlcNAc6S in BSA-GlcNAc6S_IM_ showed the highest bump thickness (**Figure 4C**).

These results showed the capacity of the GlcNAc6S to generate a cellular T-dependent response, when it is used as an immunogen, higher than that observed with BSA-GlcNAc. Although it has been reported that BSA-GlcNAc can generate T-dependent response and cross-reactivity with cardiac myosin (Malkiel et al., 2000), we validated the use of BSA-GlcNAc as control in the immunization assays used in parallel with BSA-GlcNAc6S, since it only differs in the absence of the sulfate group and because the presence of sulfates confers relevant differences to the molecule.

In summary, BALB/c mice exposed to BSA-GlcNAc6S reproduced the same specific IgG isotype profile as that obtained with C-T exposure and showed the highest DTH measurement results. The results of humoral and cellular immune responses have demonstrated that the presence of the sulfated moieties confers differences in the specificity and the immunodominance to the molecule.

#### 3.2.4 Parasitemia Counting, Mice Survival, and IFN-γ Serological Levels in BALB/c Mice Pre-Exposed to BSA-GlcNAc6S and Sublethally Challenged With *T. cruzi*

**Figure 5:**
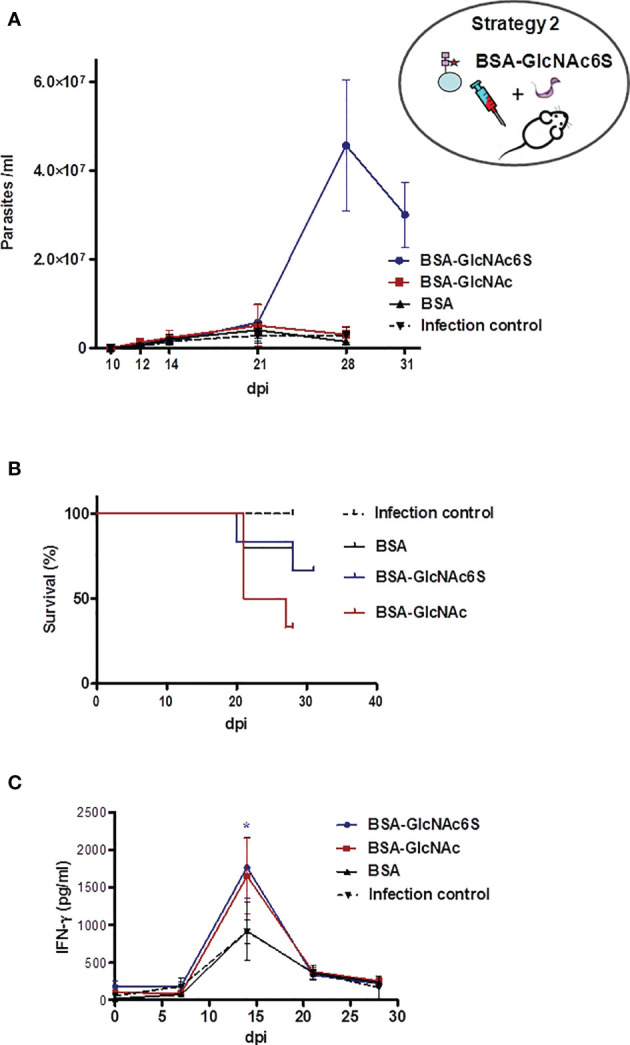
BALB/c mice immunized with GlcNAc6S-BSA and sublethally challenged with *T. cruzi*. Parasitemia, survival, and INF-γ levels during acute phase of sublethal infection from BALB/c mice, immunized with BSA-GlcNAc6S and BSA-GlcNAc, by *T. cruzi* using IFA as adjuvant. **(A)** Parasitemia (parasite number/ml of peripheral blood). **(B)** Survival percentage (%). Mice were subjected to a sublethal dose of 2,000 *T. cruzi* blood trypomastigotes, Tul 2 strain. In BSA-GlcNAc6S_IM_, parasitemia and mortality were monitored up to 31 dpi. **(C)** Production of INF-γ in BSA-GlcNAc6S_IM_ and BSA-GlcNAc_IM_. The serologic levels of IFN-γ (pg/ml) were determined by capture ELISA, after sublethal infection of nonimmunized, BSA-GlcNAc6S_IM_, BSA-GlcNAc_IM_, and BSA_IM_ in combination with IFA. The mean of each group determinations plus SD were calculated and graphed. Blue line, red line, black line, and dotted black line represent BSA-GlcNAc6S_IM_, BSA-GlcNAc_IM_, BSA_IM_, and control infection group, respectively. The oval scheme in the upper part of the figure represents strategy 2.

GlcNAc6S-BSA**_IM_** were sub-lethally challenged with *T. cruzi* Tul 2 blood trypomastigotes, showing a similar behavior to that shown by their controls (BSA-GlcNAc and BSA) up to 21 dpi. From 24 dpi + 4, about 60%–70% of mortality in BSA-GlcNAc_IM_ and about 30%–40% mice from BSA-GlcNAc6S_IM_ were registered. Subsequently, euthanasia was performed at 28 dpi (**Figure 5A**), except for BSA-GlcNAc6S_IM_ because the levels of parasitemia continued to increase. In this group, the mortality and the parasitemia were followed up to 31 dpi with the aim to register the decrease in the parasitemia curve. Surprisingly, even though mice had been subjected to a sublethal infection, they remained living, showing an exacerbated parasitemia in the order of 10^7^ parasites/ml (**Figures 5A, B** **)**. At 31 dpi, the euthanasia was performed to avoid the extension of suffering in animals. In parallel, serologic levels of IFN-γ were measured weekly, observing that at 14 dpi, the IFN-γ (pg/ml) values reached up to a maximum peak in immunized and control mice. Significant elevated similar levels of IFN-γ were observed in BSA-GlcNAc6S_IM_ and BSA-GlcNAc_IM_ with respect to those observed in the infection and immunization (BSA+IFA) of controls (**Figure 5C**). These results suggested that the immunization with the GlcNAc6S could be responsible for the resistance to parasite infection, and this resistant mechanism does not only depend on serological IFN-γ levels as shown in BSA-GlcNAc**_IM_** (**Figures 5C, B** **)**.

### 3.3 Passive Transference Treatment With IgG-GlcNAc6S: Effect on Cardiac Tissue Damage and *T. cruzi* Infection

The transference of purified IgG-GlcNAc6S (**Figure 1**) was performed to elucidate their role in the ultrastructural cardiac damage and after the sublethal challenge with blood trypomastigotes, in the infection by *T. cruzi.*

#### 3.3.1 Passive Transference of IgG-GlcNAc6S on BALB/c Mice: Ultrastructural Cardiac Tissue Damage

**Figure 6:**
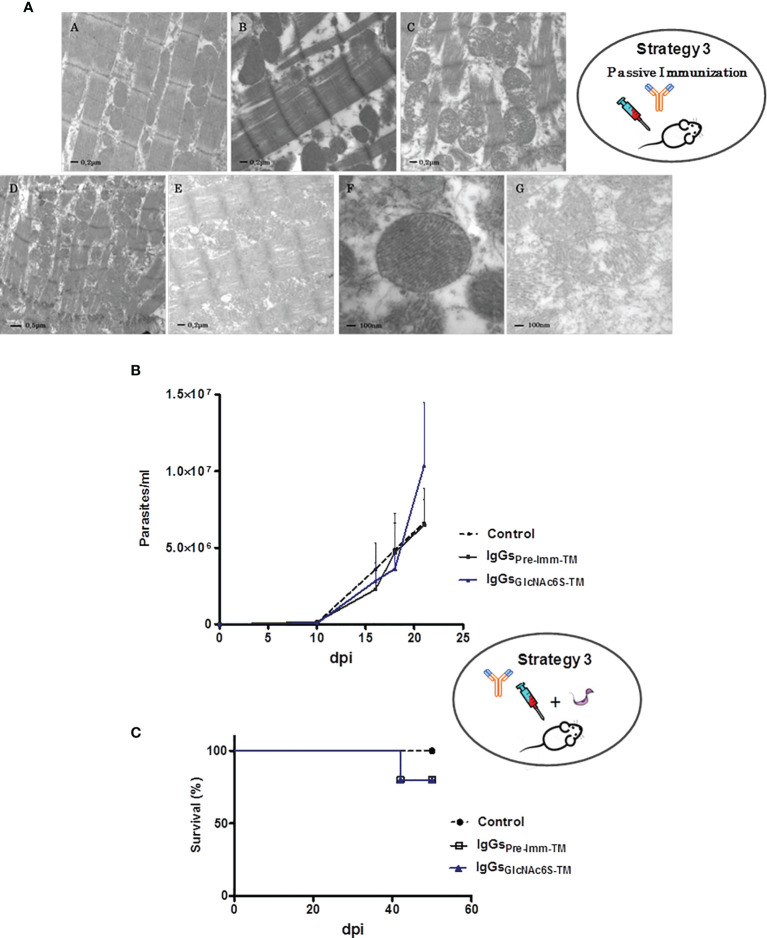
Treatment of BALB/c mice by passive transference of purified IgG-GlcNAc6S in the presence or absence of *T. cruzi* sublethal challenge. **(A)** Ultrastructural alterations of cardiac muscle tissue in mice treated with purified mouse IgG-GlcNAc6S, in the absence of infection. The ultrastructural morphologic analysis was performed by electron microscopy of transmission. Ultramicrograph of cardiac tissue from control mice (A), IgG-pre-imm-_TM_, mouse treated with purified IgG-pre-imm from mouse sera (B); cardiac tissue of mice treated with purified IgG-GlcNAc6S, IgG-GlcNAc6S-_TM_ (C–E). (A–C, E) (20,000×); in (D), (12000 ×). Mitochondria of cardiac tissue from IgG-pre-imm-_TM_ (F) or IgG-GlcNAc6S-(G). In (F) and (G), (50,000×). Acute phase of infection from BALB/c mice, treated with IgG-GlcNAc6S after a sublethal dose of *T. cruzi*. **(B)** Parasitemia (parasite number/ml of peripheral blood) and **(C)** survival percentage (%) from control of infection and treated with IgG-pre-imm and IgG-GlcNAc6S (*n* = 5 mice per group). The challenge was performed on day 1 from the experiment and the exogenous antibody transference *via* intraperitoneal administration was performed on days 2, 4, 6, 9, 12, and 17. Error bars indicate SD. Blue line, black line, and dotted black line represent IgG-BSA-GlcNAc6S-_TM_, IgG-pre-imm-_TM_, and control groups, respectively. The oval schemes in the upper and medium parts of the figure represent strategy 3 part of passive immunization and passive immunization followed by challenge with trypomastigotes, respectively.

The ultrastructural analysis of cardiac tissue from IgG-GlcNAc6S-treated mice (IgG-GlcNAc6S-_TM_), administered with IgGs purified from sera specific for BSA-GlcNAc6S, has been shown to be very interesting. The examination showed a similar disorganization to those described in C-T (Acosta et al., 2008) and GlcNAc6S-BSA_IM_. Longitudinal sections of cardiac tissue from nontreated control mice and IgG-pre-imm-treated mice (IgG-pre-imm-_TM_) that received purified IgGs from normal mouse serum both presented normal appearance. Alterations in both fibrils and mitochondria were observed in IgG-GlcNAc6S-_TM_. In **Figure 6A**.E, although the longitudinal section shows a tissue-conserved architecture, there is a decrease in the diameter of the myofibril, with debridement among myofibril components and severely altered mitochondria. Mitochondrial “swelling,” with increased space between the crests and matrix foci with cristae absence was shown. In some mitochondria, a loss in the mitochondrial membrane integrity was observed (**Figure 6A**.G). In **Figure 6A**.E, regularity and homogeneity were observed when all the sarcomeres were measured; however, the shortening of the sarcomeres observed with respect to both the control and IgG-pre-imm-_TM_ is due to the contraction state of the fiber. **Figure 6A**.C shows a notable myofibril disorganization, a prominent myofibril disruption, loss of large uniformity in sarcomeres, lack of parallel connection among myofibrils at the expense of the increase of interfibrillar sarcoplasmic space, and altered mitochondria without a characteristic alignment pattern. In addition to the abovementioned alterations, IgG-GlcNAc6S-_TM_ presented discontinuous band Z (**Figures 6A.**C**, A.**D****) with respect to the prominent and lined transversal parallel way observed in the normal tissue pattern (**Figure 6A.**A).

Despite the numerous alterations observed in the tissues of the mice treated with IgG-GlcNAc6S, the integrity of intercalary discs remained conserved (**Figure 6A**.D). The ultrastructural alterations produced by the interaction between homologous IgG-GlcNAc6S, and the mice cardiac tissue, demonstrate the harmful activity of these antibodies on the cardiac ultrastructure as we have detected in BSA-GlcNAc6S_IM_ and have previously described in C-T_IM_ (Acosta et al., 2008).

#### 3.3.2 Parasitemia Counting and Mice Survival in BALB/c Mice Sublethally Challenged and Treated With IgG-GlcNAc6S

BALB/c mice were sublethally challenged with blood trypomastigotes of *T. cruzi* Tul 2. A day after the sublethal infection, the intraperitoneal administration of purified IgG-GlcNAc6S and IgG-pre-imm began. Parasitemia was followed each 2–3 days up to day 21, which is the fourth day after the application of the last dose of IgGs. Excessively elevated parasitemia was registered only in blood of IgG-GlcNAc6S-_TM_. Control mice groups receiving vehicle or IgG-pre-imm-_TM_ registered similar parasitemia curves throughout infection, both lower than those from IgG-GlcNAc6S-_TM_. These findings demonstrate that the IgG-GlcNAc6S are involved in the interaction parasite/host during the infection by *T. cruzi* (**Figure 6B**).

Neither deaths were registered in the monitored parasitemia period nor significant differences observed in the mortality of the mice groups. Both IgG-pre-imm-_TM_ and IgG-GlcNAc6S-_TM_ groups only presented a mortality lower than 20%. However, we consider that these mice deaths were associated to infection by *T. cruzi* but not to the treatment received by BALB/c mice. This fact may be explained because it is known that the half-life of exogenous antibody transference did not usually surpass 20 days after their administration, and the deaths occurred on 42 **+** 2 dpi (**Figure 6C**) (Didoli et al., 2000; Martin et al., 2007). Once more, mice remained alive with surprising elevated parasitemias, as we showed in C-T_IM_ and BSA-GlcNAc6S_IM_, confirming that IgG-GlcNAc6S participates in parasite-host interplay favoring *T. cruzi* infection. These findings suggest that IgG-GlcNAc6S are involved in a parasite strategy capable of increasing their infectivity and, at the same time, of participating in mechanisms of resistance to maintain living to their host.

## 4 Discussion

**Figure 7:**
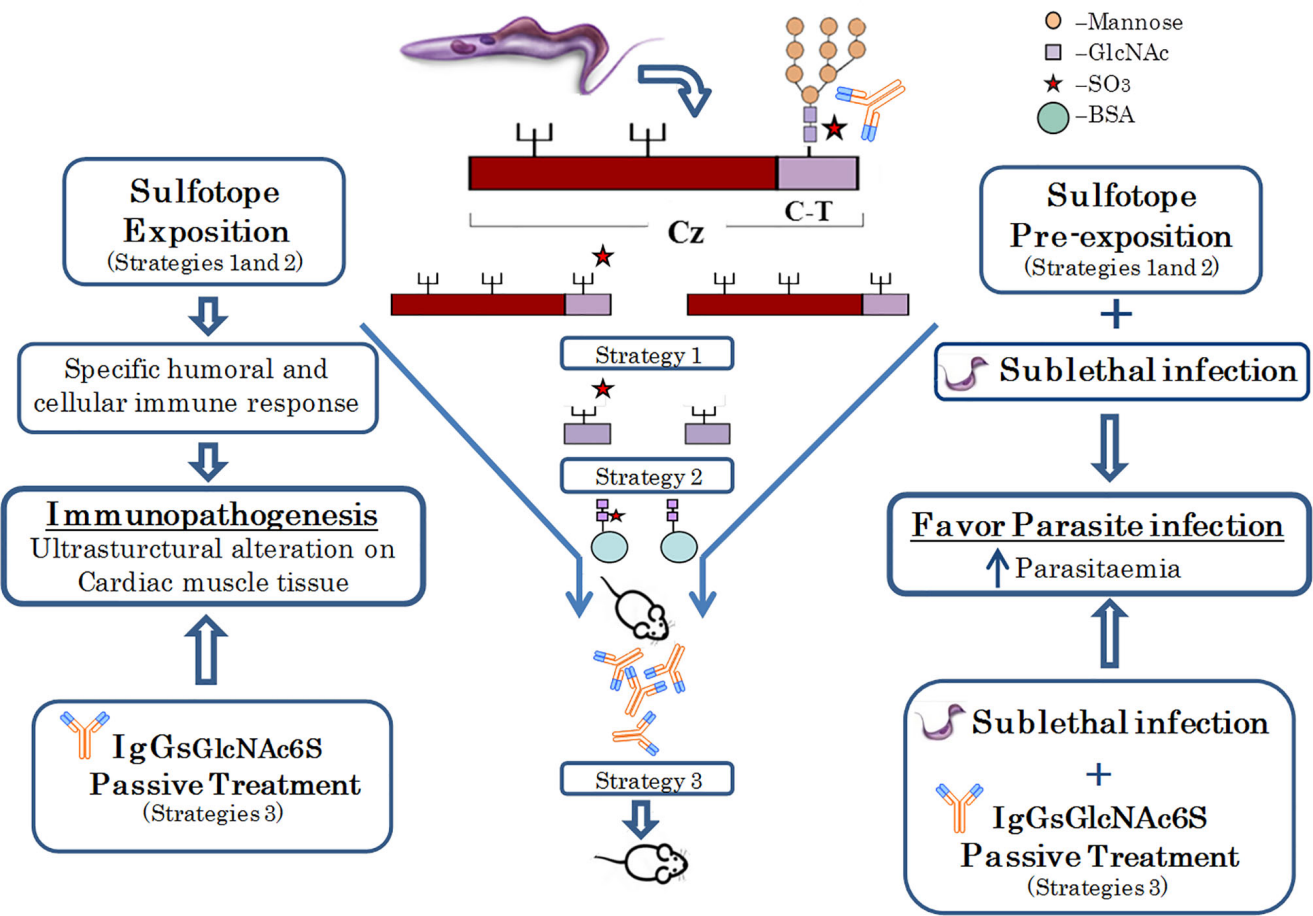
Native Cz was obtained from *T. cruzi*; C-T was purified from Cz. The immunogenic pairs Cz/dCz and C-T/dC-T were obtained by chemical desulfation process. The latter were used in strategy 1. In Strategy 2, the synthetic immunogens GlcNAc6S/GlcNAc coupled to BSA, as carrier proteins were used. Both strategies 1 and 2 evidenced both the humoral and cellular-specific immune responses and the ultrastructural pathogenesis in the cardiac muscle tissue, after *in vivo* exposition to the sulfotope. In mice, pre-exposed to the sulfotope (strategies 1 and 2) and sublethally challenged, the infection showed to be favored, with elevated parasitemias. Finally, in strategy 3, passive treatment with antibodies obtained in strategy 2 was performed. This treatment has demonstrated that IgG-GlcNAc6S, by *in vivo* exposition, have generated ultrastructural alterations in heart tissue and the administration of IgG-GlcNAc6S postsublethal challenge favored parasitic infection. 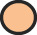, mannose; 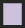, GlcNAc; 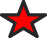, SO3; 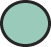, BSA; 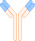, IgG-GlcNAc6S.

Cruzipain glycoprotein is a major cysteine protease and a main antigen from *Trypanosoma cruzi* (Duschak and Couto, 2009). Previous results of our research group have revealed that sulfated N-linked oligosaccharides are present in glycoproteins from trypanosomatids and that GlcNAc-6-SO_3_ from natural C-T of Cz are antigenic in the natural course of *T. cruzi* infection (Barboza et al., 2005; Acosta et al., 2008). In addition, structural analysis, and specific requirements of GlcNAc-6-SO_3_, together with the specific immune responses to these sulfated epitopes have been demonstrated (Acosta et al., 2008; Couto et al., 2012). Herein, we delved deeper into the effects of *in vivo* exposure to sulfotopes in a BALB/c murine model, mainly focusing on immune and tissue responses, in the absence of infection and in the outcome of *T. cruzi* infection, demonstrating for the first time the input of GlcNAc6S from Cz by their sulfotope-specific IgGs (IgG-GlcNAc6S) in the immunopathogenesis of experimental ChD. To elucidate the direct and/or indirect effects of GlcNAc6S on the selected mice model, a series of three independent exposition strategies were followed: (1) an immunization with purified C-T of Cz; (2) the use of a synthetic GlcNAc6S coupled to BSA as immunogen, and (3) a passive transference treatment of purified IgG-GlcNAc6S. The immunization strategies were followed by further sublethal challenge with *T. cruzi*, whereas the treatment with purified IgG-GlcNAc6S began 1 day after the sublethal challenge (**Figure 7**). Sublethal doses were selected because the immunogens were molecules involved in the tissular immunopathogenesis and the experimental design required evaluating parasitemia curves and analysis of tissues once the acute phase of the infection has been surpassed (Wood et al., 1982; Torrecilhas et al., 2009; Nogueira et al., 2015).

In this work, the selected susceptible and autoreactive strain of BALB/c mice has demonstrated to be an excellent model for immunopathogenesis process since (i) their immune response profile to *T. cruzi* infection is well known (Cardoni et al., 1999; Roggero et al., 2002); (ii) this strain has the ability of reproducing an acute and chronic phase model (Cardoni et al., 1999); (iii) this susceptible strain survives to elevated parasitemias in the acute phase (Cardoni et al., 1999; Wrightsman et al., 1982); (iv) it is a chronic model, allowing to study tissue pathogenesis in the chronic phase (Cardoni et al., 1999; Roggero et al., 2002; Soprano et al., 2018); and (v) merely, the selected female mice are more resistant to the *T. cruzi* course of infection. Even more, due to the existence of abundant previous data, the model murine-BALB/c-*T. cruzi*-Tul 2 strain was appropriately selected (Cardoni et al., 1999; Giordanengo et al., 2000a; Roggero et al., 2002; Giordanengo et al., 2002; Acosta et al., 2008; Bontempi et al., 2015; Giordanengo et al., 2020b).

It is known that BALB/c mice drive to response biased towards Th2 (Huang et al., 1997; Kuroda et al., 2002), although in *T. cruzi* infection the strain does not present as marked polarization as it was observed in Leishmaniasis. In the acute phase of the *T. cruzi* infection, the response at the splenic level, linked to the initial control of the parasite, is Th1 (Cardoni et al., 1999; Rostamian et al., 2017). Thus, cytokine hypothetical splenic profile obtained in C-T_IM_ could be ascribed to the involvement of parasite sulfotopes.

Usually, BALB/c mice strain presented high titles of humoral responses, and BALB/c mice immunization with Cz triggered an autoimmune response, myosin-binding IgGs, and cross-reactivity between parasite and host self-components (Giordanengo et al., 2000a; De Souza et al., 2001; Guiñazu et al., 2004; Giordanengo et al., 2020b). The presence of IgG deposits on the cardiac myofibrils (Giordanengo et al., 2000b), inflammatory infiltrates, and myopathic changes (Giordanengo et al., 2000a) have indicated the participation of the autoantibodies in the development of muscle-tissue damage in Cz-immunized mice. Antibodies specific for sulfated moieties, cross-reactive with various self-antigens, contained in heart (Acosta et al., 2008) and probably in other tissues, are displayed in mice immunized with sulfotope-containing antigens and subjected to the experimental *T. cruzi* infection. In this line, IgG deposits on the cardiac myofibrils in Cz-immunized mice have suggested the participation of the IgG-GlcNAc6S causing ultrastructural cardiac disturbances. Hence, the model murine-BALB/c-*T. cruzi*-Tul 2 strain has been shown to be very suitable, considering both the obtained results in conjunction with the earlier experience acquired in our laboratory.

### 4.1 BALB/c Mice Immunization With C-T: Effects on Cardiac Muscle Tissue Ultrastructure, Immune Responses, and Course of *T. cruzi* Infection

#### 4.1.1 Effects on Cardiac Muscle Tissue

Cz has been early detected in heart specimens from chronic ChD patients (Morrot et al., 1997; Scharfstein and Morrot, 1999), revealing a potential role in the immunopathogenesis and has been involved in the induction of mice target tissue damage (Giordanengo et al., 2000a; Giordanengo et al., 2000b; Giordanengo et al., 2002). However, the histopathology analysis of heart tissue from immunized mice with purified antigens, as inactive C-T or inactivated Cz, did not show any differences by optical microscopy in comparison with controls. We have demonstrated that the C-T domain is responsible for cardiac ultrastructural abnormalities in C-T_IM_, in the absence of infection (Acosta et al., 2008).

#### 4.1.2 Effects of Sulfotope Exposure to Immunized BALB/c Mice (Th bias, Cytokine Profile) and Along the Course of *T. cruzi* Infection

It is worth mentioning that the experiment design aimed to determine the involvement of the GlcNAc6S as an immunopathogenic factor. Thus, the use of IFA allowed us evidence that shows that the effect in the immune response and immunopathogenesis was due to the presence or absence of the sulfotopes, minimizing any type of immunomodulation associated to the adjuvant. IgG2a/IgG1 ratio suggested an immune response biased to Th2 against the natural molecule present in the parasite in all the immunization groups (Maassen et al., 2003; Yang et al., 2011). The evaluation of the cytokine profile of both C-T_IM_ and dC-T_IM_ was not performed under challenge with *T. cruzi* because these measurements would not allow an evaluation of the effects to only be attributed to the C-T, due to the masking produced by the multiplicity of parasite antigens. It should be noted that a quality of a Th response may predict outcome against infection (Darrah et al., 2010). In this way, the use of C-T purified from epimastigotes of *T. cruzi* as stimulus of splenocytes from immunized mice, in the absence of infection, allowed to emulate the hypothetical cytokine profile that should trigger an exposition to parasite natural GlcNAc6S-containing antigen, in C-T_IM_ and dC-T_IM_. In this sense, previous exposure to GlcNAc6S on C-T_IM_ seemed to induce the production of relevant cytokines in spleen cells to face up the challenge with *T. cruzi*, suggesting a proinflammatory/anti-inflammatory balance more favorable than that observed in the dC-T group. Some research studies support that those mice inoculations with molecules involved in the immunopathology of the disease triggered the development of severe pathologies after challenge with trypomastigotes (Wood et al., 1982; Torrecilhas et al., 2009; Nogueira et al., 2015). Then, knowing the ultrastructural alterations present in the cardiac muscle, the IgG2a/IgG1 bias, and cytokine profile in C-T_IM_, it was surprising that mice were able to become chronic, surpassing such exorbitant parasitemia levels. The C-T_IM_ response, although it was not effective in controlling parasitemia, due to sublethal doses becoming exacerbated, reflected mice resistance in the survival rates (more than 50%). In accordance with previous results (Acosta et al., 2008), dC-T_IM_ showed inability to mount a significant cellular immune response, a cytokine profile like that of the control group but with a significant IL-10 increase, under C-T stimulation and an IgG2a/IgG1 bias to Th2. Therefore, the *in vivo* exposition to the immunogen, in absence of sulfotopes would be conditioning the immune system to react in a worse way than in their presence, to course the acute phase of the infection. However, both C-T_IM_ and dC-T_IM_ were compelled to face exacerbated and lengthier parasitemia periods than the control group. *T. cruzi* surface sulfotopes have been identified in trypo and amastigote forms and have been involved in the cellular parasite infection process (Ferrero et al., 2014). Exacerbated parasitemia levels in C-T_IM_ and dC-T_IM_ evidenced parasites achieving successful infection and replication in host tissues when immune responses were unfavorably conditioned by immunization.

These immunization assays, in the presence or absence of the GlcNAc6S, followed by sublethal challenge demonstrated the role of the sulfotope taking part of *T. cruzi* virulence. Then, mice that mounted an inadequate anti-inflammatory response to the dC-T were exposed for the first time to the parasite sulfated antigens by sublethal infection, presenting elevated peaks of parasitemia levels, like C-T_IM_ and a higher score damage on skeletal muscle than C-T_IM_. It might be postulated that such virulence could be also expressed under immune-compromised conditions. The skeletal muscle has been shown to be the tissue capable of evidencing severe histopathological effects of the GlcNAc6S as “natural” virulence factor, when faced up to the challenge with *T. cruzi* postimmunization with dC-T. In this sense, we could infer that sulfotopes might be involved in the parasite skeletal muscle tropism.

The greatest number of cytokines must be evaluated to understand the mechanisms involved in the host defense when facing *T. cruzi* infection. The determination of IL-4 production, as a representative cytokine of Th2-type response, had already shown undetectable or almost negligible ELISA values in supernatant of spleen cells from Cz_IM_ (Giordanengo et al., 2002; Guiñazu et al., 2004; Frank et al., 2008). Therefore, suitable controls (Con-A) and appropriated techniques allowed us in detecting levels of IL-4, in the order of picograms per milliliter, as measured by flow cytometry, and were only detectable in mice previously exposed to C-T, using C-T (sulfotopes) as stimuli. The use of dC-T or dCz as stimuli was unable to induce IL-4 levels.

In this study, C-T_IM_ presented a mixed response (Bontempi et al., 2015; Gonzalez et al., 2013) similarly to other studies in the literature using Cz as immunogen (Frank et al., 2008). The mixed response was composed by significant levels of IL-10, IL-6, IL-4, IFN-γ, and IL-17 with respect to the dC-T_IM_ and control. By some unknown mechanism, C-T_IM_ reached to surpass excessively high and prolonged periods of parasitemia better than dC-T_IM_. Thus, it is relevant to consider the interaction among cytokines and their role in possible survival mechanisms during the acute phase. Simultaneously, C-T_IM_ induced elevated IFN-γ and IL-10 levels. The concurrent increase of both cytokines has been described in BALB/c mice infected with *T. cruzi* and it has been consistent with the described studies on cytokine responses to intracellular pathogens (Bohn et al., 1994; Pie et al., 1996), suggesting a beneficial role for IL-10, increasing the host resistance during the intracellular infection, despite their anti-inflammatory effects. Likewise, IL-10 can inhibit the production of TNF-α and counteract detrimental biological effects on the overall state of the animal once the maximum peak of parasitemia has been surpassed (Smith et al., 1994; Roggero et al., 2002), as reflected in the healthy appearance of C-T_IM_ after parasitemia. On the other hand, in *T. cruzi* infection, IL-17 has also been described as a potent inducer of the proinflammatory cytokines IL-6, TNF-α, and IFN-γ (Miyazaki et al., 2010). That report emphasizes the efficient “IL-17 host-protector” effect in the acute phase, activating the key cells for parasite destruction. In the same way, IL-6 levels stood out powerfully in C-T_IM_, being the last multifunctional cytokine involved in the regulation of the acute inflammatory reaction, in the differentiation of B cells, as well as in the activation of T cells (Van Snick, 1990; Akira et al., 1993; Truyens et al., 1994).

The most favorable response for the control of *T. cruzi* infection is associated with a Th1 profile, but due to the recognition of a T-regulatory profile and to the description of the Th17 lineage, the Th1/Th2 paradigm had to be revised, leading to the consideration of cytokines as pr-inflammatory and anti-inflammatory and to interpret cytokines, belonging to the different profiles, in accordance with the pathology considered.

Several authors have considered the use of ratios: IFN-γ/IL-10 as indicator of an IL-10-mediated regulatory response, required to avoid the detriment caused by the exacerbated response during the acute phase of *T. cruzi* infection (Jankovic and Trinchieri, 2007; Costa et al., 2009) and Th1/Treg as indicator of the tissue damage in the chronic infection (Pan et al., 1995; Gonzalez et al., 2013). Another ratio between antagonist cytokines, IL-17/IL-10, which was also used in those cases of Th17/Treg balance, has been shown to be critical (Guo, 2016). Further to the cited “host-protector” effect of the proinflammatory IL-17, this cytokine in mice experimentally infected with *T. cruzi* has been shown to be a modulator of the Th1-type response (Guedes et al., 2010). Therefore, the IL-17/IL-10 ratio represents the regulation between antagonist proinflammatory and anti-inflammatory cytokines. The significant levels of IL-10 in dC-T_IM_ lean the balance towards an anti-inflammatory regulatory profile. In turn, the elevated IL-17 levels in C-T_IM_ might participate by modulating IFN-γ and other components of Th1-type response during the acute infection and by a possible survival of IL-17-associated mechanism. It is worth noting that IL-17/IL-10 ratios were based on cytokine levels of C-T_IM_ and dC-T_IM_, in the absence of infection. However, under infection conditions, a marked difference between groups cannot be discarded, since the source of IL-17 level production is still an unanswered question. We can envisage that Th17 cells might be its origin source, but during the infection, also transialidase, and the living parasites are capable of stimulating this cytokine production by B cells through their function as lymphoid cells of innate immunity (Leon and Lund, 2013). It is worth noting that stimulation of spleen cells from C-T_IM_ with active Cz produced higher IL-17 levels than those obtained with C-T stimulation, reinforcing the speculation that IL-17 levels in challenged C-T_IM_ might be also produced by an additional cell source. Moreover, when Cz is used as stimulus, the relevance of the conformational structure adopted by this sulfotope into the complete Cz molecule should not be discarded. This inducer effect produced with Cz was not observed in dC-T_IM_. Data obtained are included in **Supplementary Figure S3**.

In summary, under C-T sulfotope exposition approaches: (i) ultrastructural cardiac alterations were the picture of the effects that caused the sulfotope or their actively produced IgG-GlcNAc6S by exposition to limited and repeated doses of C-T as immunogen and (ii) parasitemia and survival were the results of an immune response mounted previously to face up the pathogenic agent. Both effects were attributed to the same sulfotope, but they happened on different ways. Considering that the immune cross-reactivity between Cz and myosin is mainly focused on the C-T (Acosta et al., 2011), the GlcNAc6S-associated ultrastructural damage was involved in the cross-reactivity between Cz and muscular components, among them, myosin. Parasitemia, mortality, and histopathological damage postchallenge reflected the host availability of resistant mechanisms dependent of immune responses specific for GlcNAc6S, triggered by C-T or dC-T, to limit the parasite virulence. The results showed no association between host resistance to the acute phase and cardiac ultrastructure.

### 4.2 BALB/c Mice Immunization With BSA-GlcNA6S: Effect on Cardiac Ultrastructure, Immune Responses, and Course of *T. cruzi* Infection

Cardiac ultrastructure of BSA-GlcNAc6S_IM_ showed similar alterations to those observed in C-T_IM_, confirming that the sulfotope is involved in the tissue immunopathogenesis. Despite the cross-reactivity described between BSA-GlcNAc and host’s self-proteins (Malkiel et al., 2000), slightly ultrastructural abnormalities were observed in BSA-GlcNAc_IM_, suggesting that isotypes specific for BSA-GlcNAc could be contributing in a small part to the whole effect described of the complete sulfotope.

For immunization assay, we emulate the GlcNAc-6-SO_3_ located in the C-T of Cz by coupling GlcNAc6S to a protein carrier, BSA. Memory T-cell responses to BSA-GlcNAc6S, observed by intradermoreaction, have demonstrated that the sulfated molecule confers immunodominance to the GlcNAc6S (the highest measurements were obtained in BSA-GlcNAc6S_IM_ with BSA-GlcNAc6S as intradermal antigen). In addition, BSA-GlcNAc6S has demonstrated to be highly immunogenic and its humoral immune response to be intense and highly specific, reproducing the isotype profile previously obtained with C-T as immunogen. Besides, these antibodies were named IgG-GlcNAc6S due to their enrichment in the specificity for GlcNAc6S. Although humoral T-dependent response specific for BSA-GlcNAc generates cross-reactivity with cardiac myosin and proteins from the cytoskeleton, among others, has been reported (Malkiel et al., 2000), BSA-GlcNAc was used as a counterpart without sulfate to evidence the sulfated molecule effect on the immune response. Additionally, it is known that not all the oligosaccharide molecules of GlcNAc, present in the N-glycosylation site of the C-T of Cz, are sulfated (Barboza et al., 2005; Couto et al., 2012). Herein, the cross-reactivity between GlcNAc6S and GlcNAc was demonstrated. GlcNAc molecules, present at C-T glycosylation sites, can generate IgG2a-GlcNAc with low specificity of recognition.

After sublethal infection with *T. cruzi*, in BSA-GlcNAc6S_IM_, euthanasia was carried out a few days later than that from the other mouse groups. It was surprising that BSA-GlcNAc6S_IM_ survived to exorbitant parasitemias that continued rising at difference with control groups, even after a high serological IFN-γ peak was recorded.

In some way, the GlcNAc6S helps the parasite to evade the host’s immune response, to successfully infect tissues, followed by replication, reaching a high blood trypomastigote counting after being infected with a sublethal dose of *T. cruzi*. Moreover, BSA-GlcNAc6S_IM_ clearly showed that the resistance to the acute phase of the infection does not only depend on the high levels of IFN-γ, signaling the requirement of an adequate global immune response.

In addition, using this second strategy, ultrastructural cardiac damage, a specific humoral and cellular immune response and exacerbated parasitemias associated to the GlcNAc6S exposition were observed. So far, both immunization approaches: strategy 1 (C-T) and strategy 2 (BSA-GlcNAc6S), have evidenced the involvement of the GlcNAc6S and/or their IgG-GlcNAc6S in the ultrastructural immunopathogenesis of heart tissue and in the experimental infection by *T. cruzi*. However, none of the two strategies allowed elucidating whether the observed effects were attributed to the GlcNAc6S and/or to their IgG-GlcNAc6S.

### 4.3 Passive Transference Treatment With IgG-GlcNAc6S: Effect on Cardiac Tissue Damage and *T. cruzi* Infection

The role of IgG-GlcNAc6S in the immunopathogenesis and in *T. cruzi* infection was determined by passive transference of IgG-GlcNAc6S. The injuries observed in mice that passively received IgG-GlcNAc6S have demonstrated that IgG-GlcNAc6S are responsible for causing ultrastructural cardiac damage. Surprisingly, in all mice subjected to the sublethal challenge with *T. cruzi* blood trypomastigotes followed by IgG-GlcNAc6S, an excessively high parasitemia was registered without mortality records.

#### 4.3.1 The Role of Antibodies Specific for GlcNAc6S in the Immunopathogenesis

The evaluation of the humoral immune response to sulfated moieties in the C-T was mainly IgG2b, demonstrating that IgG2b reactivity was completely abolished when desulfated antigens were used as immunogens. Binding of IgG2b antibodies to host tissues may be responsible for the tissue damage observed in sulfated C-T of Cz_IM_. By contrast, the lack of tissue damage in dC-T_IM_ was associated to the absence of IgG2b response (Acosta et al., 2008). Interestingly, evidence about IgG2b specific for sulfatides in sera from rats experimentally infected with *T. cruzi*, which bind to homologous neural tissue was described as predominant isotypes of these “autoantibodies” specific for sulfatides. Thus, it was proposed that the recognition of sulfo-cerebrosides of cellular surface by these specific rat “auto-antibodies” might play some detrimental role *in vivo* (Avila, 1992; Avila et al., 1998; Didoli et al., 2000; Acosta et al., 2012). It is important to highlight that IgG2b isotype is capable of fixing the complement or mediate cellular cytotoxicity reactions (Feldman et al., 1999). On the other hand, a correlation between IgG2a and IgG2b with autoimmunity processes has also been described (Giordanengo et al., 2000b). In this line, isotypes specific for BSA-GlcNAc6S, IgG2a, and IgG2b, with high affinity for sulfated molecules, suggest a possible association between the sulfo-specific isotypes and the cross-reactivity with host self-proteins. It might be postulated that IgG2a and IgG2b could exert its detrimental role on cardiac ultrastructure by cross-reactive antibodies fixing to myosin and/or to other components of muscle tissue, through the consequent complement activity or antibody-dependent cytotoxicity (ADCC) (Lopez et al., 1981; Tambourgi et al., 1989; Feldman et al., 1999; Nimmerjahn and Ravetch, 2005; Baudino et al., 2008; Gutierrez et al., 2009).

#### 4.3.2 The Role of IgG-GlcNAc6S Along the Course of *T. cruzi* Infection

Mice were infected with sublethal doses the day before the beginning of the treatments with IgG-GlcNAc6S, IgG-pre-imm, and control IgGs. The end point of passive transference assay was set at day 21, because until that moment, the source of antibodies was almost exclusively exogen. Lastly, the highest parasitemia record, in the order of 1 × 10^7^ parasites/ml, was registered in IgG-GlcNAc6S-_TM_. It was surprising to obtain such a high parasite level when using lower sublethal doses (Lu et al., 2008) than those used in strategies 1 and 2. Thus, it confirmed the input of IgG-GlcNAc6S favoring the infection by *T. cruzi*, with parasitemia levels that probably reflected the multiplication of the replication cycles in the affected tissues. In addition, similarly to the results obtained in the preceding approaches, it was notable that mice treated with IgG-GlcNAc6S could surpass very high parasitemia counts. In this case, that resistance could not be ascribed to Th1/Th2/Treg/Th17 cytokine balance triggered by immunization and only depended on natural resistance of BALB/c mice model to high parasitemias.

While the control group did not record deaths, the mortality rate found in treated mice, fewer than 20% close to 40 dpi, could be due to individual intragroup variability. IgG-GlcNAc6S are highly represented by the described opsonizing isotype, IgG2b. It could be postulated for any of the three strategies addressed that IgGs with high affinity for GlcNAc6S play a role in host/parasite interaction collaborating with the parasite in cellular invasion. Masking GlcNAc6S of parasite surface plus phagocytosis mediated by opsonization through their Fc domains could be a mechanism exerted by IgG-GlcNAc6S. On the other hand, IgG-GlcNAc6S without an opsonizing capacity could also play a masking role for GlcNAc6S with evasion of a strong specific response, similar to fabulation, mechanism described in *T. cruzi* (Bontempi and Cazzulo, 1990; Beresain et al., 2003).

Both humans and mice IgGs are glycoproteins highly N-glycosylated and bear a lot of GlcNAc (Arnold et al., 2007; de Haan et al., 2017). Likewise, not all GlcNAc on the N-glycosylation sites on the C-T of Cz are sulfated. Thus, IgG2a-GlcNAc with low specificity generated during *T. cruzi* infection could participate in an evasion mechanism like those described in bacteria that produce glycosidases, blocking IgGs functions in phagocytosis mediated by opsonization (Alhorn et al., 2008).

IgG-GlcNAc6S have been demonstrated to play a dual role, favoring infection and participating in cardiac ultrastructural pathogenesis. Herein, we can confirm indirect sulfotope effects, ascribed to IgG-GlcNAc6S, in the immunopathogenesis of experimental ChD. Ongoing assays in the course in our research laboratory will allow elucidating whether GlcNAc6S also play a direct role in the infection and in the pathogenicity mechanism in experimental ChD.

## 5 Conclusion

In summary, the pre-exposition of the sulfotopes by immunization of BALB/c mice with C-T and BSA-GlcNAc6S has demonstrated their input in the immunopathogenesis because of the ultrastructural alterations, displayed on cardiac muscle tissues, and favored the parasite infection, as reflected by the high parasitemias reached. Both active immunizations have revealed the involvement of the GlcNAc6S in some mechanisms of resistance to elevated parasitemias, possibly associated to the cytokines profile. Moreover, GlcNAc6S have been proven to be an immunodominant antigen capable of triggering strong immune responses and of generating high levels of humoral responses enriched in IgG-GlcNAc6S. Furthermore, we could envision that GlcNAc6S might either boost up virulence factors for *T. cruzi* infection or play a part in parasite infection strategies or in evasion mechanisms.

After applying the first and second strategies, having actively exposed the GlcNAc6S to BALB/c mice, either the ultrastructural cardiac muscle tissue damage or the outcome of *T. cruzi* infection in favor of the parasite, showed to be consistent with a direct and/or indirect effect of the GlcNAc6S or their IgG-GlcNAc6S, respectively. Nevertheless, the third strategy using a passive transference of IgG-GlcNAc6S, allowed us to confirm the indirect role of the GlcNAc6S, through their IgG-GlcNAc6S, in the immunopathogenesis and in the infection by *T. cruzi*

As a striking feature, we have demonstrated that IgG-GlcNAc6S are responsible for causing a severe ultrastructural cardiac damage, probably by inducing complement or ADCC and can favor the parasite infection by opsonizing or masking the surface molecules, as shown by the high parasitemia values obtained. Interestingly, IgG-GlcNAc6S could play a role in mice survival, arresting parasites in target tissues by blockage of sulfated virulent factors and/or by taking part in the restriction of the infection. On top of this, the effects of IgG-GlcNAc6S were identified, playing a role both in the pathogenesis and in the infection process.

## Data Availability

The original contributions presented in the study are included in the article/**Supplementary Material**. Further inquiries can be directed to the corresponding author.

## Ethics Statement

All experimental procedures including animal handling and experimental design were conducted in accordance with guidelines for care and use for laboratory animals established and approved by the Review Board of the Ethics Committee from the National Institute of Parasitology “Dr. Mario Fatala Chaben”, ANLIS-Malbrán, Ministerio de Salud. RENIS Register Number, 000028.

## Author Contributions

VD, LS, GG, and ME contributed to the design of the study. LS, MF, GG, ML, and ME acquired the data. VD, LS, MF, and GG helped with the analyses and interpretation of the data. VD and LS wrote the manuscript. All authors contributed to the article revision and approved the submitted version.

## Funding

Funding was provided by grants provided by Agencia Nacional de Investigaciones Científicas y Técnicas (ANPCyT), PICT: 2006-00145; Consejo Nacional de Investigaciones Científicas y Técnicas (CONICET), PIP: 11220170100150CO, and Universidad de Buenos Aires (UBA): 21020180600771BA. LS was supported by a fellowship from ANPCyT. MF and LS were supported by CONICET fellowships. MF was supported by a fellowship from Mundo Sano Foundation in all cases, during the development of this work.

## Conflict of Interest

The authors declare that the research was conducted in the absence of any commercial or financial relationships that could be construed as a potential conflict of interest.

## Publisher’s Note

All claims expressed in this article are solely those of the authors and do not necessarily represent those of their affiliated organizations, or those of the publisher, the editors and the reviewers. Any product that may be evaluated in this article, or claim that may be made by its manufacturer, is not guaranteed or endorsed by the publisher.

## Abbreviations

Cz, cruzipain; C-T, C-terminal domain; GlcNAc-6-SO_3_, *N*-acetyl d-glucosamine-6-sulfate; GlcNAc6S, GlcNAc-6-SO_3_, sulfated epitopes, “sulfotopes”; GlcNAc, *N*-acetyl d-glucosamine molecules; IFA, incomplete Freund adjuvant; C-T_IM_, C-T-immunized mice; dC-T_IM_, dC-T-immunized mice; BSA-GlcNAc6S, synthetic GlcNAc-6-SO_3_ coupled to BSA; BSA-GlcNAc, GlcNAc coupled to BSA; BSA-GlcNAc6S_IM_, BSA-GlcNAc6S-immunized mice; BSA-GlcNAc_IM_, BSA-GlcNAc-immunized mice; IgG-GlcNAc6S, IgG specific for sulfotopes or sulfo-specific antibodies; IgG-pre-imm, IgGs from sera of preimmune mice; IgG2-SO_3_, human IgG2 antibodies specific for sulfated structures; IgG2a-GlcNAc, IgG2a specific for GlcNAc; IgG-GlcNAc6S-_TM_, IgG-GlcNAc6S-treated mice; IgG-pre-imm-_TM_, IgG-pre-imm-treated mice; BSA-GlcNAc6S_(ID)_, BSA-GlcNAc6S intradermal antigen; BSA-GlcNAc_(ID)_, BSA-GlcNAc intradermal antigen; BSA_(ID)_, BSA intradermal antigen; DTH, delayed-type hypersensitivity.
