# Supplementary material for: Cruzipain Sulfotopes-Specific Antibodies Generate Cardiac Tissue Abnormalities and Favor Trypanosoma cruzi Infection in the BALB/c Mice Model of Experimental Chagas Disease
Source: Front Cell Infect Microbiol. 2022 Jan 4;11:814276. doi: 10.3389/fcimb.2021.814276 (PMC8763857; doi:10.3389/fcimb.2021.814276)
Supplement: Supplementary file 1 [file DataSheet_1.docx]

Sup Figure 1


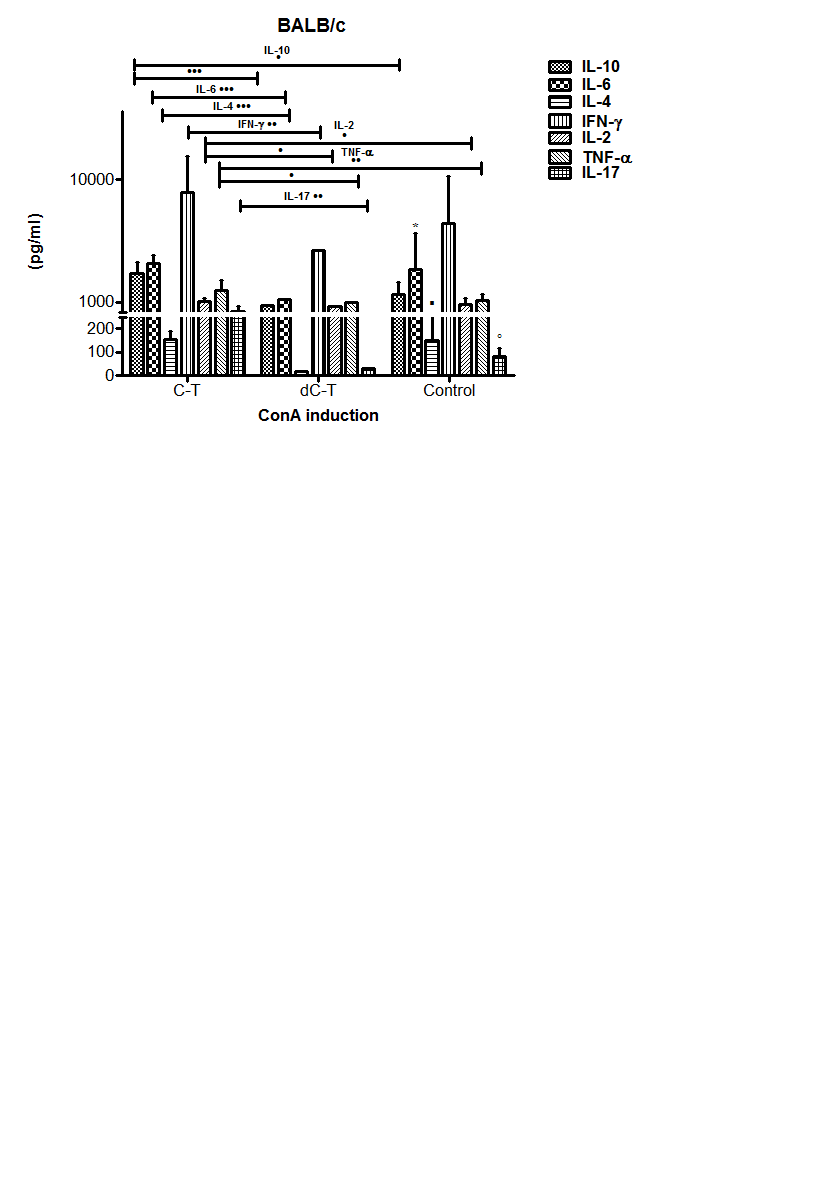


**Supplementary Figure 1**. **Flow cytometry measurement of the cytokine levels:** of IL-10, IL-6, IL-4, IFN-γ, IL-2, TNF-α and IL-17 in supernatants of spleen cells cultures from C-T_IM_, dC-T_IM_ and control mice, stimulated with ConA. The supernatants were obtained after 72hs of stimulation with ConA (2 μg/ml). The concentration of each cytokine (pg/ml) was obtained with the software BD FCAP Array v3.0. The bars represent the average of six determinations by duplicate with their SD. Production of IL-10: ● vs. control; ●●● vs dC-T_IM_. Production of IL-6: ●●● vs. dC-T_IM_; * vs. dC-T_IM_ (P<0.01)_._ Production of IL-4: ●●● vs. dC-T_IM;_ ■ vs. dC-T_IM_ (P<0.01). Production of IFN-γ: ●● vs. dC-T_IM_. Production of IL-2: ● vs. control; ● vs dC-T_IM_. Production of TNF-α: ●● vs. control; ● vs dC-T_IM_. Production of IL-17: ●● vs. dC-T_IM; °_ vs. dC-T_IM_ (P<0.05) _._ (● P<0.05; ●● P<0.01; ●●●P<0.001). The non-parametric Kruskal Wallis test followed by multiple comparisons Dunn’s test was used for statistical data analysis**.**

Sup Fig 2
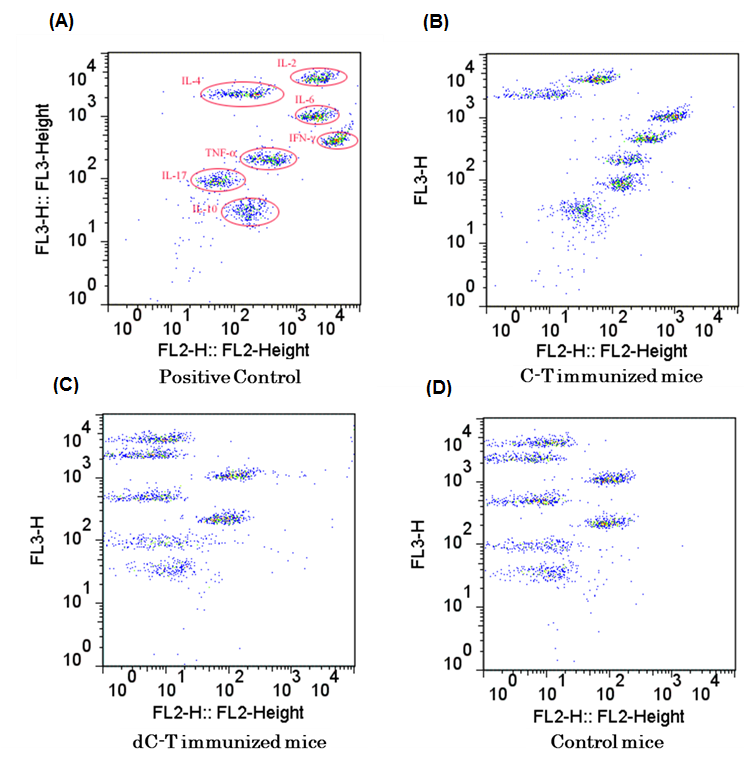


**Supplementary Figure 2. Two-dimensional dot plot corresponding to the cytokines profile of a mouse representative of each immunization group**. Positive control: supernatant of spleen cells from control mouse stimulated with Con-A + Capture beads for specific cytokines. The ellipses identify the 7 populations of beads in accordance to their fluorescence in FL-3/FL-2. C-T_IM_: supernatant of spleen cells of C-T_IM_, stimulated with C-T. dC-T_IM_: supernatant of spleen cells of dC-T_IM_, stimulated with C-T. Non-immunized control mouse: supernatant of spleen cells of non-immunized control mouse (immunized with PBS + IFA) and stimulated with C-T.

**Sup Fig 3**


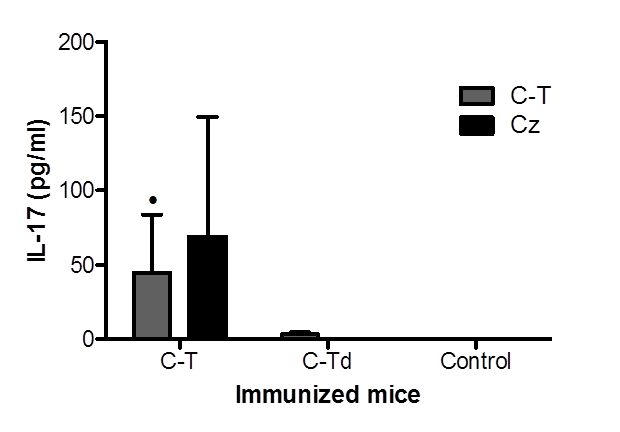


**Supplementary Figure 3. Determination of IL-17 cytokine by flow cytometry stimulated with Cz and C-T.**

Levels of IL-17 in pg/ml, measured in supernatants of cultures from spleen cells from C-T_IM_ compared to dC-T_IM_ and non-immunized control mice. The spleen cells were stimulated with Cz and C-T. ● vs. dC-T_IM_ stimulated with C-T. Bars represent the average of 6 determinations by duplicate and their SD. The statistical analysis was performed by ANOVA of two factors and test of Bonferroni for comparisons. ● (P<0.05).

Sup Fig 4


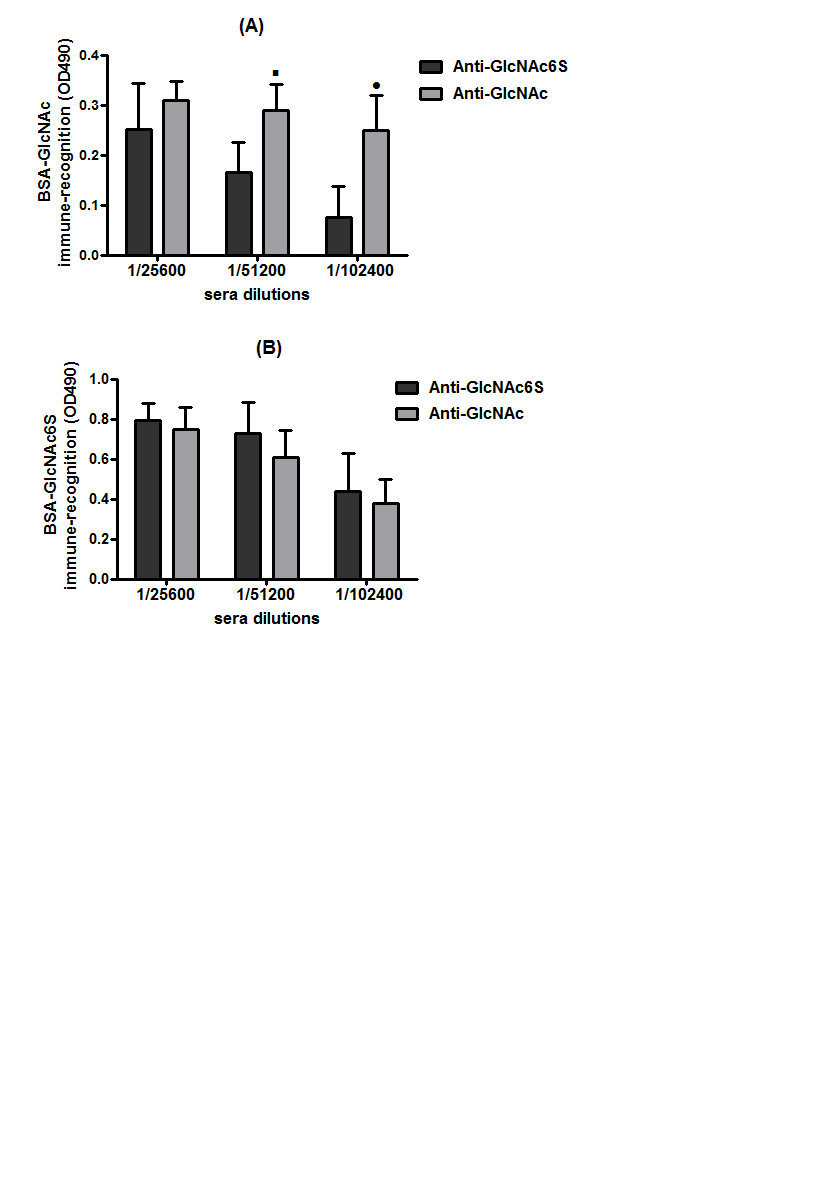


**Supplementary Figure 4. Cross-reactivity between BSA-GlcNAc6S and BSA-GlcNAc.** Immunorecognition of BSA-GlcNAc **(A)** and BSA-GlcNAc6S **(B)** by total IgGs purified from sera of mice immunized with BSA-GlcNAc6S and BSA-Glc*N*Ac by indirect ELISA. Bars represent the average of duplicate determinations and their SD. The results are representative of three similar independent experiments. ■ P<0.01 vs. anti-GlcNAc6S (1.51200); ● P <0.001 vs anti- GlcNAc6S (1:102.400). Mann Whitney test was used for statistical analysis.
